# Phytochemical alkaloids orchestrate immunometabolism against viral infections

**DOI:** 10.1093/nsr/nwaf190

**Published:** 2025-06-16

**Authors:** Cuiqin Cheng, Yao Wang, Han Wang, Meiqi Zhang, Qiqi Li, Bing Xu, Lingdong Kong, Xia Liu, Yanli Yu, Yuting He, Yingjie Chu, Zhe Liu, Yuanyuan Qiao, Xinxin Yuan, Xin Jia, Anlong Xu

**Affiliations:** School of Life Science, Beijing University of Chinese Medicine, Beijing 100029, China; Beijing Research Institute of Chinese Medicine, Beijing University of Chinese Medicine, Beijing 100029, China; School of Chinese Materia Medica, Beijing University of Chinese Medicine, Beijing 100029, China; School of Life Science, Beijing University of Chinese Medicine, Beijing 100029, China; School of Life Science, Beijing University of Chinese Medicine, Beijing 100029, China; School of Chinese Materia Medica, Beijing University of Chinese Medicine, Beijing 100029, China; School of Chinese Materia Medica, Beijing University of Chinese Medicine, Beijing 100029, China; School of Life Science, Beijing University of Chinese Medicine, Beijing 100029, China; School of Chinese Materia Medica, Beijing University of Chinese Medicine, Beijing 100029, China; School of Chinese Materia Medica, Beijing University of Chinese Medicine, Beijing 100029, China; School of Life Science, Beijing University of Chinese Medicine, Beijing 100029, China; School of Chinese Materia Medica, Beijing University of Chinese Medicine, Beijing 100029, China; School of Chinese Materia Medica, Beijing University of Chinese Medicine, Beijing 100029, China; School of Chinese Materia Medica, Beijing University of Chinese Medicine, Beijing 100029, China; School of Chinese Materia Medica, Beijing University of Chinese Medicine, Beijing 100029, China; School of Life Science, Beijing University of Chinese Medicine, Beijing 100029, China

**Keywords:** cholesterol metabolism, immunometabolism, bis-benzylisoquinoline alkaloids, NPC1-STING interface, virus, antiviral immunity

## Abstract

The role of cholesterol metabolism in antiviral immunity has been established, but if and how this cholesterol-mediated immunometabolism can be regulated by specific small molecules is of particular interest in the quest for novel antiviral therapeutics. Here, we first demonstrate that NPC1 is the key cholesterol transporter for suppressing viral replication by changing cholesterol metabolism and triggering the innate immune response via systemic analyses of all possible cholesterol transporters. We then use the Connectivity Map (CMap), a systematic methodology for identifying functional connections between genetic perturbations and drug actions, to screen NPC1 inhibitors, and found that bis-benzylisoquinoline alkaloids (BBAs) exhibit high efficacy in the inhibition of viral infections. Among all potent BBAs that we tested, tetrandrine (Tet) is the most effective, by directly binding to NPC1 and inducing lysosomal cholesterol accumulation in order to resist viral entries. Through the NPC1-STING interface mechanism, Tet further blocks STING lysosomal degradation which leads to boosting of the interferon-based antiviral response against multiple viruses both *in vitro* and *in vivo*. Therefore, BBAs represent very promising drug compounds for this newly discovered antiviral mechanism by targeting the NPC1-STING interface via cholesterol-mediated immunometabolism, which in turn disrupts the virus life cycle and boosts antiviral immunity.

## INTRODUCTION

Cholesterol metabolism generates vital membrane components and metabolites that play crucial roles in viral synthesis and assembly, also serving as a facilitator for viral entry [1,2]. Disrupting cholesterol metabolism, either through changes in intracellular cholesterol levels or distribution, directly affects the viral life cycle. Cholesterol transporters like Niemann-Pick C1 (NPC1) and the low-density lipoprotein receptor (LDLR) act as viral entry receptors, making them potential therapeutic targets for viral infections [3,4]. Furthermore, emerging evidence suggests a critical link between innate immunity and cholesterol metabolism [5]. Upon viral infection, cholesterol synthesis is decreased and accompanied by activation of the DNA sensing pathway, which leads to the upregulation of antiviral gene expression, including type I interferon (IFN-I) and interferon-stimulated genes [6]. IFN receptor (IFNAR) stimulation enhances the expression of genes like cholesterol-25-hydroxylase (*CH25H*) [7,8], which convert cholesterol to 25-hydroxycholesterol (25-HC) to block viral entry and disrupt lipid rafts, preventing viral release [8,9]. Meanwhile, these cholesterol metabolism–related ISGs reduce cholesterol synthesis to cooperatively control viral infection [10]. Consistent with this concept, genetic or pharmacological inhibition of the host cholesterol synthesis attenuates viral and microbial infections in both preclinical and clinical systems [5,6].

Lysosomes orchestrate cholesterol sensing, utilization, and recycling through synchronized interplay between hydrolytic enzymes, cholesterol transporters and signaling factors, which together enable the coordination of cholesterol metabolism with other organelles and specific gene regulation programs [11]. NPC1 is a lysosomal and endosomal membrane protein that exports cholesterol derived from low-density lipoprotein (LDL) from late endosomes and lysosomes to other cellular compartments and plays a crucial role in maintaining cholesterol homeostasis between lysosomes and the cytoplasm [12]. Niemann-Pick disease type C (NPC), a rare genetic disorder caused by *NPC1* and *NPC2* mutations, disrupts lipid trafficking and causes neurological decline [13]. Previous studies have shown that filovirus membrane fusion and escape require NPC1, beyond its cholesterol transport role [3,14]. Recent research has highlighted NPC1’s crucial role in mammalian infections by the baculovirus AcMNPV. NPC1 mediates the fusion of viral and host cell membranes, releasing viral particles from endosomes [15]. Notably, NPC1 acts as a cofactor in STING trafficking, a key component in the DNA-sensing pathway, presenting a therapeutic target to block STING degradation and enhance antiviral immunity [16]. Therefore, small molecules targeting this mechanism can disrupt the virus life cycle and enhance antiviral immunity, offering potential for antiviral drug design.

*Stephania tetrandra* and other related species of Menispermaceae serve as the main source of BBAs [17]. The plant is extensively referenced in the *Chinese Pharmacopoeia* for treating asthma, tuberculosis, dysentery and hyperglycemia in traditional Chinese medicine (TCM) [18]. Known as calcium channel blockers, BBAs have shown effectiveness against SARS-CoV-2, Ebola, and herpes simplex virus 1 (HSV-1) without toxicity in preclinical trials [19–21]. However, their broad-spectrum antiviral mechanisms against both RNA and DNA viruses remain poorly understood.

By conducting short interfering RNA (siRNA) screens, we found that the knockdown of cholesterol transport genes suppressed viral replication, with NPC1 being the most impactful. NPC1 loss or inhibition resulted in lysosomal cholesterol accumulation, similar to interferon-treated cells. Therefore, we identified genes altered by NPC1 knockdown and used the Connectivity Map (CMap) to identify small molecules that inhibit NPC1. This led to the identification of BBAs, such as berbamine (BBm), cepharanthine (Cep) and Tet, with Tet showing the strongest NPC1 inhibition. Four BBAs significantly inhibited vesicular stomatitis virus (VSV) and influenza virus A (H1N1) replication, with Tet exhibiting optimal and comprehensive antiviral activity. We found that Tet directly binds to NPC1 and blocks its cholesterol transport, causing lysosomal cholesterol accumulation that directly inhibits viral entry and initiates IFN-based antiviral innate immune response via the NPC1-STING axis. Our findings present well-demonstrated examples of pharmacological targeting on the NPC1-STING interface, critical immunometabolic targets, for improvement in blocking viral infections.

## RESULTS

### Loss of NPC1 or NPC1 pharmacological inhibition resists viral infection

**Figure 1.**
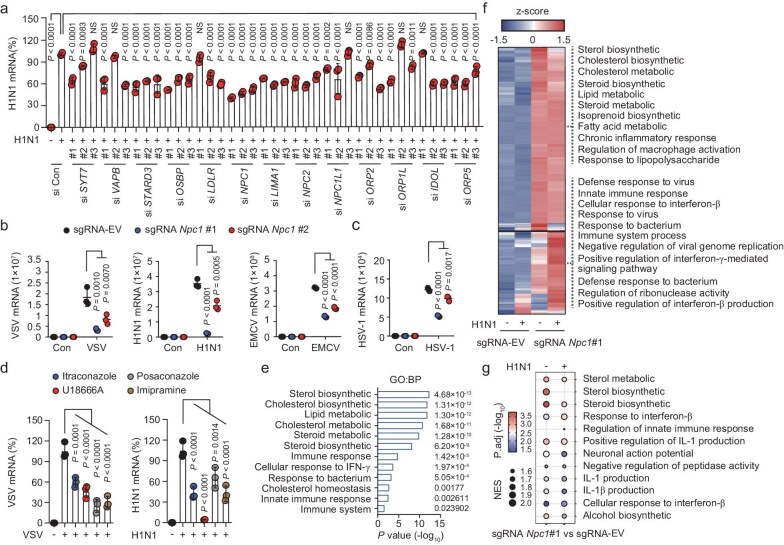
Loss of NPC1 or drug inhibition resists viral infection. (a) H1N1 mRNA expression in HepG2 cells transfected with siRNAs before infection. (b, c) Viral mRNA expression in sgRNA EV, *Npc1*#1, and *Npc1*#2 cells infected by VSV (b), H1N1 (b), EMCV (b), or HSV-1 (c). (d) Viral mRNA expression in A549 cells pretreated with itraconazole, posaconazole, U18666A, and imipramine before viral infection. (e) GO enrichment analysis of DEGs in sgRNA *Npc1*#1 vs EV cells. (f) Heatmap of DEGs in sgRNA *Npc1*#1 vs EV cells with or without H1N1 infection. (g) Dot plot of pathways in sgRNA *Npc1*#1 vs EV cells with or without H1N1 infection using GSEA. The dot size is related to the statistical significance. For a–d, *n* = 3 independent experiments. All data are presented as mean ± SD. *P* values were calculated using one-way ANOVA. NS, not significant.

Genetic or pharmacological inhibition of host cholesterol metabolism can attenuate viral infections [5,6]. To better understand the impact of host-virus interactions on cholesterol metabolism and identify potential therapeutic targets for viral infection, we performed an RNAi knockdown screen on cholesterol transport factors, which showed no toxicity in HepG2 cells after 72 hours (Fig. S1a–b). Knockdown of genes such as *SYT7, VAPB, STARD3, OSBP, LDLR, NPC1, LIMA1, NPC2, NPC1L1, ORP2, ORP1L, IDOL* and *ORP5* decreased H1N1 and VSV replication to varying extents, indicating that interfering with cholesterol metabolism via cholesterol transport could provide broad antiviral benefits (Fig. 1a, Fig. S1c).

Specifically, *NPC1* knockdown showed the most pronounced inhibition of viral replication (Fig. 1a, Fig. S1c). Using CRISPR-dCas9-KRAB, we created two *Npc1*-knockdown cell lines (sgRNA-*Npc1*) that showed a marked reduction in VSV, H1N1, encephalomyocarditis virus (EMCV) and HSV-1 replication compared to wild-type cells (sgRNA-EV) (Fig. 1b, c, Fig. S1d). NPC1 inhibitors, like itraconazole, posaconazole, U18666A and imipramine, effectively blocked VSV and H1N1 replication (Fig. 1d). This suggests that NPC1 deletion or inhibition can reduce viral replication, making NPC1 a promising target for antiviral drug development.

To investigate the mechanisms underlying NPC1-mediated antiviral effects, RNA sequencing (RNA-seq) identified 619 differentially expressed genes (DEGs) in sgRNA-*Npc1* cells compared to controls (Table S1). Gene ontology (GO) enrichment analysis and gene set enrichment analysis (GSEA) of the DEGs revealed significant enrichment in sterol biosynthesis and immune responses with or without H1N1 infection (Fig. 1e–g). To explore NPC1’s role in antiviral immunity *in vivo*, we analyzed publicly available transcriptome data from WT and *Npc1* KO mice cerebellum (GSE20450) [22]. We found significant upregulation of innate immunity genes (*Ifit2, Ifi27, Oas1a, Ccl2, Ccl5* and *Isg15*) in *Npc1* KO mice (Fig. S1e). These findings suggest that NPC1 may be a promising therapeutic target for viral infections, via the recently-identified mechanism in connecting cholesterol metabolism with innate immune responses.

### BBAs inhibit viral replication

**Figure 2.**
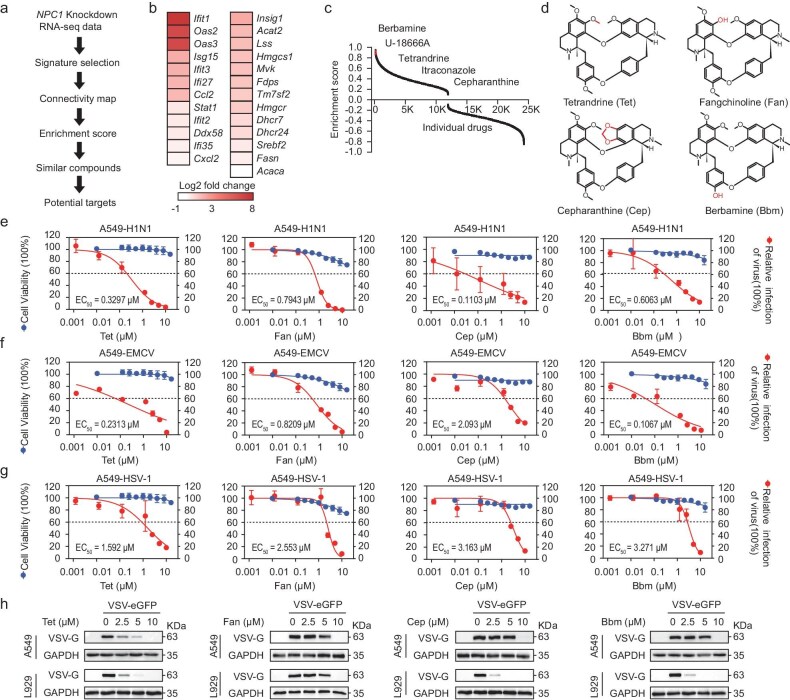
Bis-benzylisoquinoline alkaloids inhibit viral replication. (a) Schematic of CMap screening. (b) Heatmap of 25 representative genes in CMap associated with type I interferon and cholesterol metabolism pathway (sgRNA *Npc1*#1 vs EV cells). (c) Distribution of enrichment score of compounds in CMap database. (d) Four drugs: Tet, Fan, Cep, and Bbm. (e–g) Dose-response curves of infectivity (red) and cell viability (blue) in A549 cells treated with Tet, Fan, Cep, or Bbm during H1N1 (e), EMCV (f), and HSV-1 (g) infection. (h) Immunoblotting of VSV-G protein in A549 and L929 cells co-incubated with VSV-eGFP and Tet, Fan, Cep, or Bbm. For e–g, *n* = 3 independent experiments. All data are presented as mean ± SD. *P* values were calculated using one-way ANOVA.

CMap provides a systematic methodology for identifying NPC1 inhibitors by analyzing transcriptional changes and functional similarities between physiological states following *Npc1* knockdown. Using 25 representative upregulated genes linked to cholesterol metabolism and innate immunity as transcriptional profiles, we queried CMap and successfully identified U18666A and itraconazole, validating the efficiency of this approach (Fig. 2a–c). Notably, BBAs like Bbm, Cep and Tet, with similar structures and reported antiviral activity, ranked highly (Fig. 2c, d). Therefore, we propose that BBAs may be developed as new antiviral drugs based on the newly-defined therapeutic target NPC1 with the recently-identified mechanism of cholesterol-mediated immunometabolism in inhibiting viral replication.

To evaluate BBAs’ antiviral potential, we conducted assays with representative compounds (Tet, fangchinoline (Fan), Cep, Bbm) focusing on cytotoxicity, virus yield and infection rates against H1N1, EMCV, VSV and HSV-1. Cytotoxicity was evaluated in A549 and L929 cells, and viral mRNAs were measured using qRT-PCR. Tet showed the strongest antiviral effects, with EC_50_ values of 0.3297 µM (H1N1), 0.2313 µM (EMCV) and 1.592 µM (HSV-1) in A549 cells, and 0.5406 µM, 0.9892 µM and 0.1315 µM in L929 cells (Fig. 2e–g, Fig. S2a–c). Immunoblotting further revealed that Tet, Fan, Cep and Bbm inhibited VSV replication (Fig. 2h). Tet also effectively reduced H1N1 NP protein expression without causing cytopathic effects (Fig. S2d, e). These findings indicate that BBAs exhibit broad-spectrum antiviral activity, with Tet performing most effectively. Thus, our findings indicate that BBAs possess pharmacological properties comparable to *NPC1* knockdown or pharmacological inhibition, suggesting their potential as novel antiviral drugs targeting NPC1 to inhibit viral replication.

### Tet inhibits viral replication without affecting viral attachment and independently of its calcium channel-blocking activity

We then assessed Tet’s antiviral effects using a time-of-drug-addition assay (Fig. S3a), revealing that pre-treatment, co-treatment and post-treatment with Tet effectively suppressed VSV, H1N1 and EMCV infections (Fig. S3b–d). Furthermore, we found Tet inhibited VSV, H1N1 and EMCV infections during entry or post-entry stages, without affecting virus attachment (Fig. S3e–m).

A previous study showed that Ebola virus entry into host cells depends on two-pore calcium channels (TPCs) that can be disturbed by Tet, we then explored the relationship between Tet’s antiviral mechanism and TPCs using CRISPR-Cas9 to delete *TPCN1* and *TPCN2* in A549 cells [20] (Fig. S4a). Results showed Tet suppressed VSV and H1N1 replication in both WT and *TPCN* knockout cells, indicating that its antiviral activity against VSV and H1N1 is independent of TPCs (Fig. S4b, c). Given Tet’s role as a calcium channel blocker, we further investigated its antiviral activity dependence on Ca^2+^ [23]. Tet’s antiviral efficacy remained unchanged in calcium-free conditions or with nimodipine treatment, suggesting its action is independent of calcium channels (Fig. S4d–f). These findings suggest that Tet exhibits new antiviral mechanisms and targets independent of TPCs and Ca^2+^.

### Tet induces lysosomal cholesterol accumulation to inhibit virus entry

**Figure 3.**
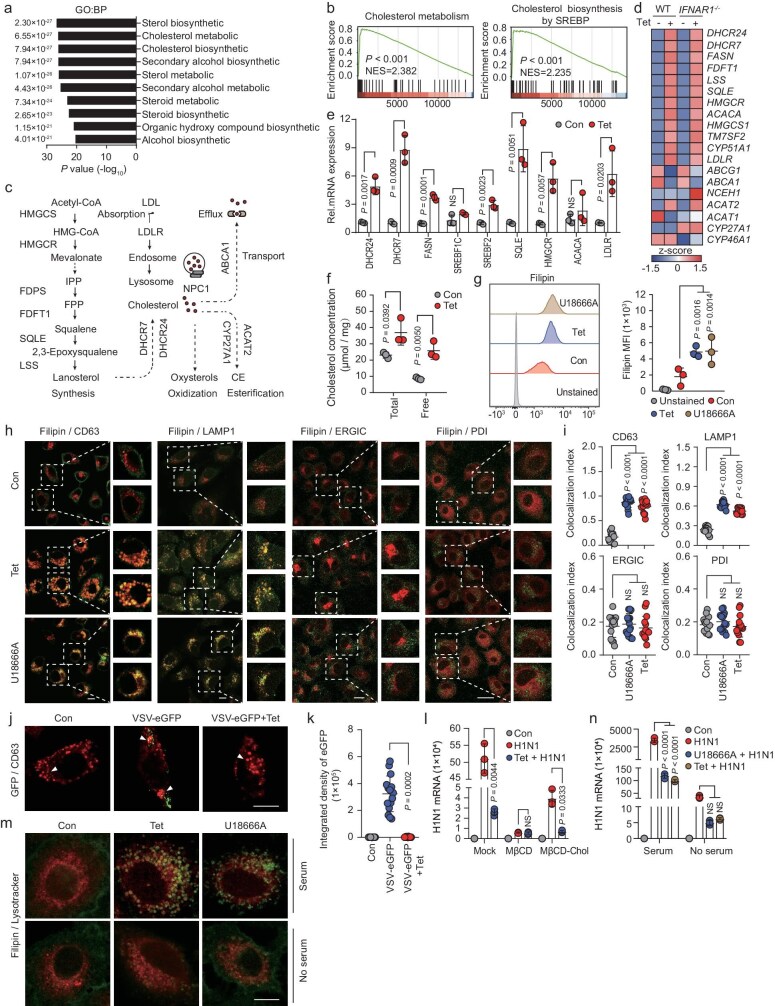
Tet inhibits viral entry by inducing lysosomal cholesterol accumulation. (a) GO enrichment analysis of upregulated genes in Tet-treated A549 cells. (b) GSEA of gene expression profile in Tet-treated A549 cells. (c) Diagram of cholesterol metabolism. (d) Heatmap in WT and *IFNAR1*^−/−^ A549 cells with or without Tet treatment. (e) Cholesterol metabolism–related gene expression in Tet-treated A549 cells. (f) Total and free cholesterol levels in Tet-treated A549 cells. (g) Flow cytometry analysis of membrane cholesterol in Tet/U18666A-treated A549 cells. (h) Confocal analysis of CD63, LAMP1, ERGIC-53, PDI, and cholesterol-staining agent filipin in Tet/U18666A-treated A549 cells, scale bar: 50 μm. (i) Quantification of co-localization in (h); *n* = 15 cells/condition. (j) Confocal analysis of CD63 and GFP in A549 cells incubated with Tet and VSV-eGFP for 1.5 h during viral entry, scar bar:10 μm. (k) Quantification of integrated density of GFP in (j); *n* = 15 cells/condition. (l) H1N1 mRNA expression in control cells, MβCD (cellular cholesterol removed) or MβCD-cholesterol (with extra cholesterol compensation) treated A549 cells with Tet followed by H1N1 infection. (m) Confocal analysis of lysotracker and filipin in Tet/U18666A-treated A549 cells under normal/serum-free medium, scale bar: 10 μm. (n) A549 cells were pretreated with normal/serum-free medium and then incubated with Tet or U18666A during H1N1 infection. For e, f, g, h, j, l, n, *n* = 3 independent experiments. All data are presented as mean ± SD. *P* values were calculated using unpaired two-tailed Student’s *t-*test (e, f, g, i) or one-way ANOVA (k, l, n). NS, not significant.

RNA-seq was conducted to profile transcriptome-wide changes in A549 cells following treatment with Tet, an effective antiviral against VSV, H1N1, EMCV and HSV-1, which showed significant changes in cholesterol metabolism pathways (Fig. 3a, b). Tet increased expression of genes involved in cholesterol synthesis (*SREBF2, HMGCR, SQLE, SREBP1C, DHCR24, DHCR7* and *FASN*) and absorption (*LDLR*). However, it did not affect the expression of genes related to cholesterol transport (*ABCA1* and *ABCG1*), oxidation (*CYP27A1*and *CYP46A1*) or esterification (*ACAT1* and *ACAT2*) (Fig. 3c–e). Supporting these findings, biochemical and flow cytometry analyses indicated higher intracellular cholesterol levels after Tet treatment (Fig. 3f, g).

The intracellular cholesterol distribution has been reported as a potential target for the disruption of ‘virus-containing cargos’, providing new avenues to combat viral infections [24,25]. Tet altered cholesterol distribution, shifting it from the plasma membrane to endosomal and lysosomal compartments, evident by colocalization with CD63 (endosomal/lysosomal marker), LAMP1 (lysosomal marker), PDI (endoplasmic reticulum marker) and ERGIC-53 (ER-Golgi intermediate compartment marker) (Fig. 3h, i). Importantly, similar lysosomal cholesterol accumulation was observed with U18666A treatment.

Previous studies show that cholesterol-loaded endosomes and lysosomes inhibit viral entry by blocking fusion. Similarly, we found that Tet blocked VSV-eGFP entry into lysosomes (Fig. 3j, k). Methyl-β-cyclodextrin (MβCD, a cholesterol-depleting agent) pretreatment reduced lysosomal cholesterol and abolished Tet’s or U18666A’s antiviral effect on VSV entry, while cholesterol supplementation using MβCD-cholesterol restored inhibition (Fig. 3l). Under serum-free conditions, both lysosomal cholesterol accumulation and Tet’s or U18666A’s antiviral effect on H1N1 entry were diminished (Fig. 3m, n). These findings suggest Tet induces cholesterol accumulation in lysosomal compartments, ultimately inhibiting viral entry.

### Tet initiates STING-dependent innate antiviral immune response

**Figure 4.**
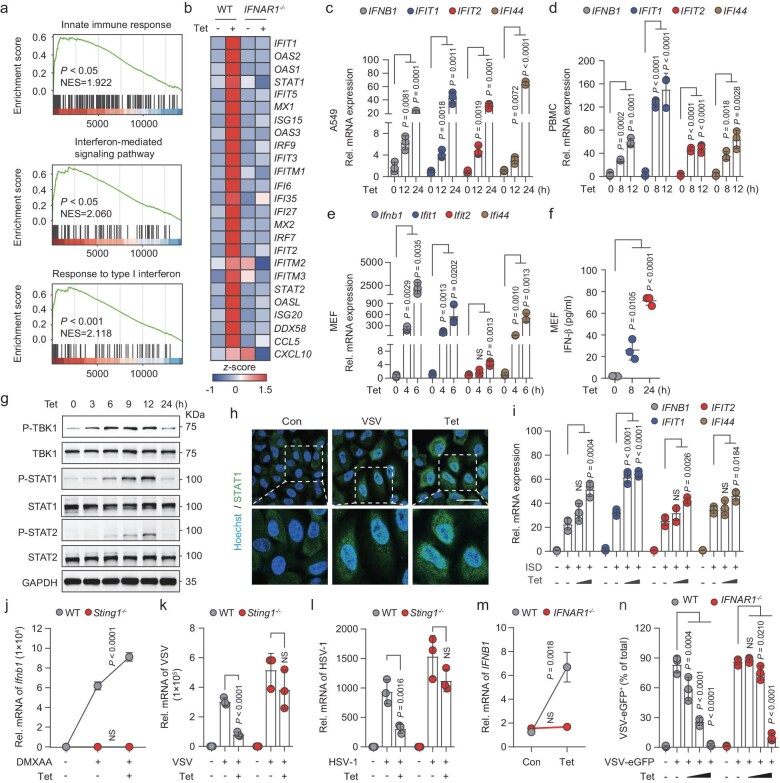
Tet activates STING-dependent antiviral innate immune responses. (a) GSEA of the gene expression profile in Tet-treated A549 cells. (b) Heatmap of ISGs in Tet-treated WT and *IFNAR1*^−/−^ A549 cells. (c–e) *IFNB1, IFIT1, IFIT2* and *IFI44* mRNA expression in A549 (c), PBMC (d) and MEF (e) cells stimulated with Tet. (f) IFN-β secretion in Tet-treated MEF cells. (g) Immunoblotting of p-TBK1, TBK1, p-STAT1, STAT1, p-STAT2 and STAT2 proteins in Tet-treated THP-1 cells. (h) Confocal analysis of nuclear localization of STAT1 in Tet-treated A549 cells, scale bar: 50 μm. (i) *IFNB1, IFIT1, IFIT2* and *IFI44* mRNA expression in THP-1 cells transfected with ISD followed by Tet. (j) *IFNB1* mRNA expression in WT or *Sting*^−/−^ MEF cells stimulated with DMXAA followed by Tet. (k, l) VSV (k) or HSV-1 (l) mRNA expression in WT or *Sting^−^^/^^−^* BMDM (k) or MEF (l) cells co-treated with Tet and viruses. (m) *IFNB1* mRNA expression in Tet-treated WT or *IFNAR1*^−/−^ A549 cells. (n) Flow cytometry analysis in Tet-treated WT or *IFNAR1*^−/−^ A549 cells infected with VSV-eGFP. For c, d, e, f, h, i, j, k, l, m, n, *n* = 3 independent experiments. All data are presented as mean ± SD. *P* values were calculated using unpaired two-tailed Student’ s *t-*test (c, d, e, f, m) or one-way ANOVA (i, j, k, l, n). NS, not significant.

We explored how Tet inhibits viral replication post-entry and its effects on the immune response compared to *NPC1* loss-of-function. GSEA revealed a significant enrichment of pathways related to the innate immune response and interferon response in Tet-treated A549 cells (Fig. 4a). Tet upregulated ISGs like *IFIT1, IFIT2* and *ISG15*, but this induction was absent in IFNAR1-deficient cells (Fig. 4b). qRT-PCR analysis further revealed the upregulation of *IFNB1* and several ISGs in Tet-treated cells (Fig. 4c–e). Tet also induced IFN-β production and increased the phosphorylation of TBK1, STAT1 and STAT2 in a time- and dose-dependent manner (Fig. 4f, g, Fig. S5a, b). Confocal imaging confirmed Tet promoted STAT1 nucleus translocation, initiating IFN expression (Fig. 4h). In agreement with the data above, Tet increased *Ifnb1* and ISGs mRNA levels *in vivo*, particularly in lungs and livers (Fig. S5c–f), consistent with its selective accumulation in mouse lung [26].

STING is crucial for the host to eliminate invading viruses and to initiate the expression of type I IFN. Studies have shown that NPC1 serves as a co-factor in STING trafficking, which enhances STING-dependent immunity [16]. Based on the similarity of the Tet phenotype to NPC1 gene silencing (Fig. 2c), we hypothesize it activates STING-dependent immunity. We stimulated THP-1 cells with ISD to activate the STING-dependent antiviral immune pathway and observed that Tet significantly increased *IFNB1* and ISGs gene expression (Fig. 4i). Similarly, Tet increased *Ifnb1, Ifi44, Ccl5* and *Cxcl10* gene expression in STING agonist DMXAA treated WT MEF cells, whereas their expression was abolished in *Sting^−^^/^^−^* MEF cells (Fig. 4j, Fig. S5g, h). The antiviral effects of Tet were markedly reduced in *Sting*^−/−^ cells, supporting its role in inducing STING-dependent innate antiviral immunity (Fig. 4k, l). The antiviral effect and boosted ISGs of Tet were significantly attenuated in *IFNAR1^−^^/^^−^*A549 cells, indicating that Tet activates the antiviral immune response in an IFN-based manner (Fig. 4m, n, Fig. S5i, j). Interestingly, IFN-β treatment induced lysosome cholesterol accumulation, similar to Tet and U18666A (Fig. S5k). Our study demonstrated that Tet initiates STING-dependent IFN-based innate antiviral immunity after virus entry, boosting ISGs and suppressing viral replication.

### BBAs orchestrate cholesterol metabolism and antiviral immune response

Given the similar chemical structures of BBAs like Fan, Cep and Bbm, we hypothesized they would induce similar transcriptional and functional changes. We tested this by analyzing drug-induced transcriptome changes in A549 cells. GO enrichment analysis and GSEA revealed highly similar gene expression patterns among Tet, Fan, Cep and Bbm, particularly in cholesterol metabolism and antiviral immunity (Fig. S6a–e). Interestingly, drug-upregulated cholesterol metabolism genes were enriched in both WT and *IFNAR1^−^^/^^−^* A549 cells, whereas antiviral response genes were enriched only in WT cells, with Tet and Fan triggering higher expression of antiviral immune-related genes (Fig. S6f). Pathway analysis further confirmed enhanced cholesterol metabolism and antiviral immune responses in WT cells but not in *IFNAR1^−^^/^^−^* cells (Fig. S6g). These results suggest BBAs share a common mechanism that modulates cholesterol metabolism and antiviral immunity to inhibit viral replication.

### Tet targets the NPC1/STING axis to resist virus infection

**Figure 5.**
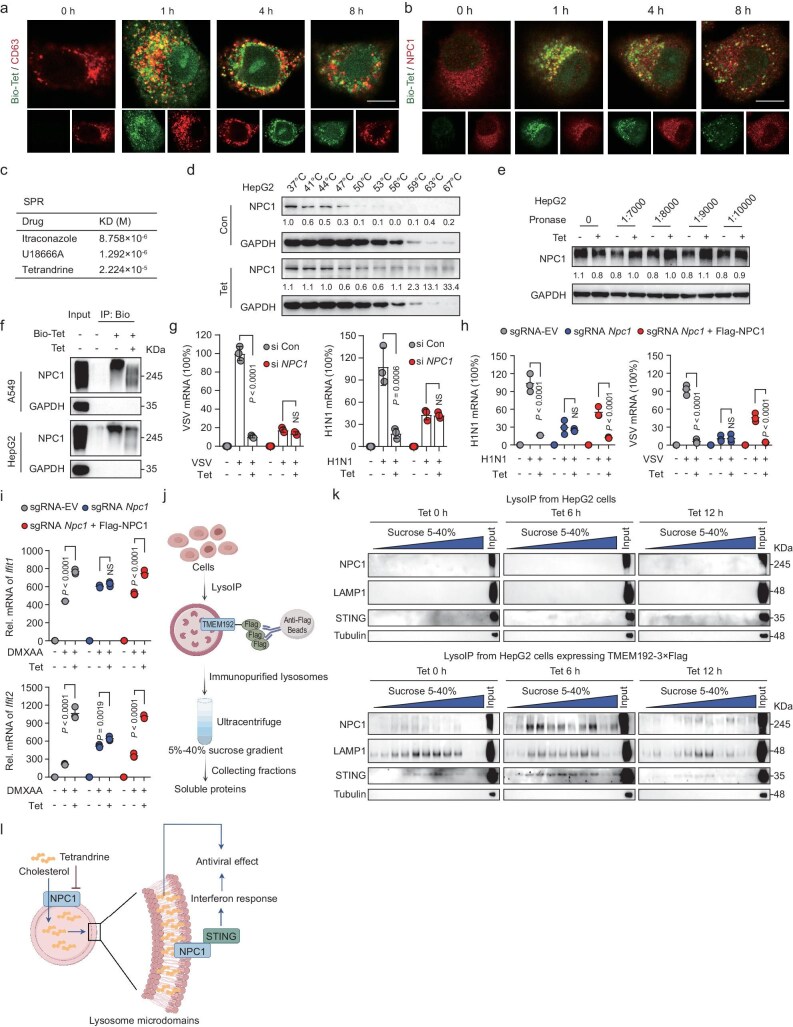
The dual functions of Tet to resist viral entry and activate antiviral immunity through the NPC1/STING axis. (a, b) Confocal analysis of Bio-Tet with CD63 (a) or NPC1 (b) in A549 cells, scale bar: 10 μm. (c) Binding affinity analysis of NPC1 with itraconazole, U18666A, or Tet was determined by SPR. (d, e) CETSA (d) and DARTS (e) analysis of NPC1 with Tet in HepG2 cells. (f) Streptavidin-covered beads with biotin, Bio-Tet, Bio-Tet + Tet were incubated with cell lysates. As indicated, the levels of NPC1 protein in bound proteins (IP) and total proteins (Input) were determined using immunoblotting. (g) Viral mRNA expression in si*NPC1*-transfected cells treated with Tet. (h) Viral mRNA expression in sgRNA-EV, sgRNA *Npc1*, and sgRNA *Npc1* cells transfected with Flag-NPC1 treated with Tet. (i) *Ifit1* and *Ifit2* mRNA expression in sgRNA-EV, sgRNA *Npc1*, and sgRNA *Npc1* cells transfected with Flag-NPC1 stimulated with DMXAA and Tet. (j) Schematic of LysoIP-DEG. (k) Immunoblotting analysis of the indicated proteins in LysoIP-DEG in Tet-treated HepG2 cells. (l) Schematic model of NPC1/STING axis-mediated viral inhibition by Tet. The schematic diagram (j) and model (l) were created by Figdraw. For a, b, g, h, i, *n* = 3 independent experiments. All data are presented as mean ± SD. *P* values were calculated using one-way ANOVA (g, h, i). NS, not significant.

To reveal the molecular mechanisms of how Tet resists viral entry and initiates antiviral immune responses, we used biotin-labeled Tet (Bio-Tet) to investigate its intracellular distribution (Fig. S7a). Confocal imaging showed Bio-Tet accumulated in lysosomes within an hour, declining over time, consistent with lysosomes’ role in virus degradation (Fig. 5a, Fig. S7b–d) [11]. Additionally, flow cytometry and qRT-PCR analysis confirmed Bio-Tet’s antiviral activity and its role in inducing antiviral response and cholesterol metabolism–related genes (Fig. S7e, f). CMap analysis identified Tet as a top NPC1 inhibitor, similar to itraconazole and U18666A (Fig. 2c). Subsequent experiments showed Tet induces lysosomal cholesterol accumulation, resembling NPC1-silenced cells (Fig. S7g). Based on these findings, we hypothesize that Tet exerts its pharmacological function through NPC1.

To confirm this function, we conducted confocal microscopy to reveal the co-localization of Bio-Tet with NPC1. Results showed a temporal pattern of increase and then decrease over time (Fig. 5b, Fig. S7h). Further, molecular docking confirmed strong binding affinities of Tet, Fan, Cep and Bbm to the SSD and NTD domains of NPC1 (Fig. S7i). Surface plasmon resonance (SPR) confirmed the specific binding of Tet to NPC1, with a dissociation constant (KD) of M (2.224 × 10^−5^) (Fig. 5c). Subsequently, cellular thermal shift assay (CETSA) and drug affinity responsive target stability (DARTS) experiments showed Tet enhanced NPC1 stability against degradation (Fig. 5d, e Fig. S7j). Pull-down analysis further validated Bio-Tet’s interaction with NPC1, which was competitively inhibited by untagged Tet (Fig. 5f).

To investigate the role of the Tet-NPC1 interaction in antiviral effects, we employed RNAi to knockdown NPC1 in HepG2 cells, finding that NPC1 knockdown diminished Tet’s antiviral effects against VSV and H1N1 (Fig. 5g, Fig. S7k, l). In sgRNA-*Npc1* cells, Tet’s antiviral effects were attenuated but restored with Flag-tagged NPC1 reintroduction (Fig. 5h, Fig. S7m). Tet and U18666A showed similar antiviral effects during virus entry and post-entry, but not during attachment (Fig. S7n). Taken together, these results suggest that Tet binds to NPC1 and confers resistance to viral infection.

Since NPC1 functions as a cofactor for STING trafficking, it interacts with STING and facilitates STING recruitment to the lysosome for degradation [16]. We hypothesize that Tet selectively inhibits STING degradation to enhance antiviral immunity through the NPC1/STING axis. Notably, Tet significantly attenuated the time-dependent degradation of STING induced by DMXAA, thereby prolonging TBK1 and STAT1 activation, similar to BafA1 (Fig. S7o). Tet treatment–enhanced *Ifit1* and *Ifit2* expression was observed in sgRNA-EV cells, but this effect was abolished in NPC1-deficient cells and restored in sgRNA-*Npc1* cells transfected with Flag-NPC1 (Fig. 5i). These observations support the initiation of IFN-based innate antiviral immunity and the antiviral effect of Tet in an NPC1/STING-dependent manner.

We then immunopurified lysosomes using density gradient ultracentrifugation (LysoIP-DEG) to examine the fraction of lysosomal microdomains after Tet treatment (Fig. 5j, Fig. S7p). HepG2 cells transfected with the TMEM192-3 × Flag showed higher LAMP1 expression, indicating more lysosomal microdomains than controls (Fig. 5k). Notably, Tet enhanced NPC1 and STING expression in lysosomal microdomains after 6 hours, suggesting it affects the NPC1/STING axis in lysosomes (Fig. 5k). These data indicate that Tet-NPC1 interaction inhibits lysosomal cholesterol transport, inducing lysosomal microdomains that promote antiviral immunity through clustering of the NPC1/STING axis (Fig. 5l).

### Tet inhibits virus infection *in vivo*

**Figure 6.**
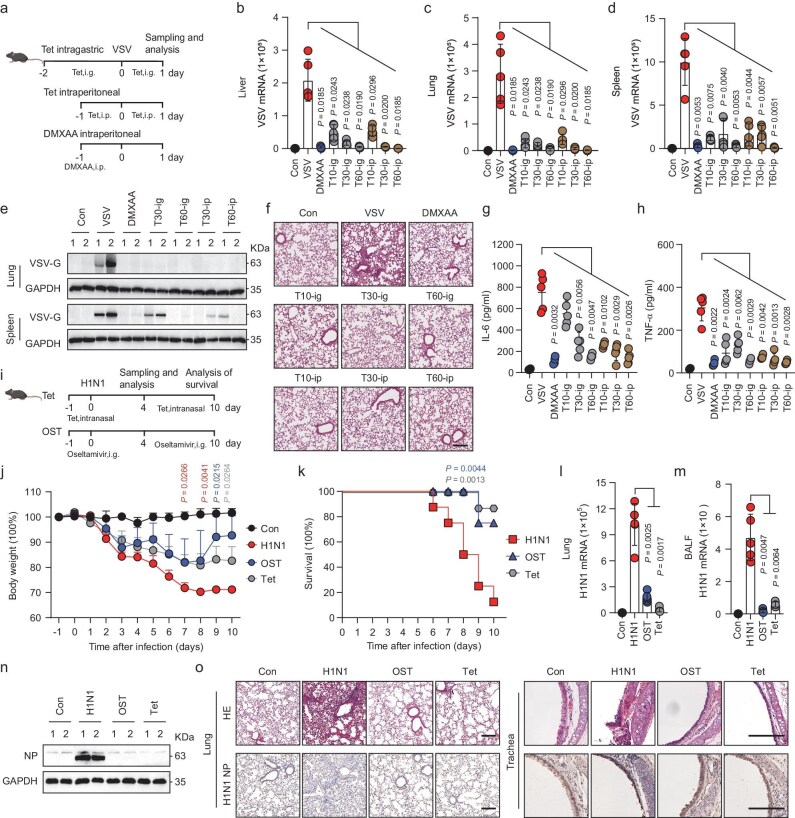
Tet inhibits VSV and H1N1 virus replication *in vivo*. (a) Schematic of VSV infection mouse model. (b–d) VSV mRNA expression in mouse liver (b), lung (c) and spleen (d) tissue (*n* = 5). (e) VSV-G protein levels in mouse lung and spleen tissue. (f) H&E staining of inflammatory infiltration in mouse lung tissue, scale bar: 200 μm. (g, h) IL-6 (g) and TNF-α (h) secretion in mouse serum (*n* = 5). (i) Schematic of H1N1 infection mouse model. (j) Changes in body weight of mice infected with H1N1 (*n* = 8). (k) Survival curves of mice. (l, m) H1N1 mRNA expression in mouse lung tissues (l) and BALF (m) (*n* = 5). (n) NP protein changes in mouse lung tissue. (o) H&E staining of inflammation in mouse lung tissue (scale bar: 200 μm) and tracheae (scale bar: 100 μm). Immunohistochemistry (IHC) of NP protein particle in mouse lung tissue (scale bar: 200 μm) and tracheae (scale bar: 100 μm). All data are presented as mean ± SD. *P* values were calculated using one-way ANOVA. NS, not significant.

Pharmacokinetic profiling reveals that Tet exhibits a high volume of distribution with low systemic clearance, supporting its broad tissue accumulation [26]. Aligning with clinical silicosis regimens (60–100 mg TID), we administered Tet via intraperitoneal and intragastric routes (10–60 mg/kg) 24 hours pre-VSV infection (Fig. 6a) [27]. DMXAA, a STING agonist, served as a positive control. Tet significantly reduced VSV genome copies in liver, lung, and spleen tissues (∼0.5–1 log₁₀) and inhibited VSV-G protein expression in lungs and spleens (Fig. 6b–e). Histological examination (H&E) staining revealed Tet reduced lung pathology and cellular infiltrations (Fig. 6f). Tet and DMXAA markedly decreased IL-6 and TNF-α levels in serum (Fig. 6g, h). These findings suggest Tet protects against VSV by reducing viral replication and inflammation in mice.

Concurrently, Tet was tested against H1N1 *in vivo*, with oseltamivir as a control (Fig. 6i). Intranasal administration of Tet expedited body weight recovery and improved survival rates, akin to oseltamivir (Fig. 6j, k). Tet markedly reduced H1N1 genome copies in lung tissue and bronchoalveolar lavage fluid (BALF) by about one log10 and inhibited NP protein expression, similar to oseltamivir (Fig. 6l–n). Tet significantly alleviated alveolar damage and inflammatory infiltrations in lung tissues as shown by H&E staining (Fig. 6o). Collectively, these results confirm Tet’s ability to suppress H1N1 replication and mitigate associated inflammatory responses in mice.

## DISCUSSION

BBAs, such as Bbm, Cep and Tet, are Food and Drug Administration (FDA) or National Medical Products Administration (NMPA)-approved compounds in the U.S. and China for conditions like silicosis, arthritis and radioactive leukopenia [28]. Since the SARS-CoV-2 outbreak, they have gained attention for their antiviral properties against viruses *ex vivo* and *in vivo* like SARS-CoV-2, Ebola and HSV-1 [20,21,29]. Mechanistically, Bbm disrupts the endolysosomal trafficking of ACE2 and decreases its concomitant expression on the cell surface, preventing SARS-CoV-2 entry. Tet blocks two endosomal calcium channels and prevents Ebola virus entry through endosomal compartments [20]. Cep promotes autophagy and directly degrades viral particles to inhibit HSV-1 replication [21]. Given the similar chemical structures of BBAs, the mechanisms above do not fully account for the broad-spectrum protection provided by BBAs against RNA and DNA viruses. Exploring the detailed molecular mechanisms and potential therapeutic targets through host-virus interactions could explain the broad-spectrum antiviral activity of BBAs and for the better development of compounds as antiviral drugs.

One striking finding of our study is the identification in which these BBAs target NPC1 specifically, leading to reversible lysosomal cholesterol accumulation similar to that in interferon-treated cells. Notably this accumulation, induced by BBAs or interferons, differs from the irreversible accumulation of cholesterol sphingomyelin and other lipids in lysosomes, which is characteristic of NPC lysosomal storage disease [13,30]. Therefore, the subcellular phenotype of lysosomal cholesterol accumulation represents important chemobiological and immunological functions involved in host cell defense against pathogen infections and may serve as a better chemobiological target for combating viral infectious diseases.

Here, we demonstrated that Tet’s antiviral effect relies on lysosomal cholesterol accumulation induced by inhibiting the cholesterol transporter NPC1. Removing intracellular cholesterol eliminates lysosomal accumulation and attenuates Tet’s antiviral effect, while adding external cholesterol restores its antiviral effect. This is consistent with previous studies that cholesterol accumulation in the late endosomal and lysosomal compartments impairs viral entry of IAV, VSV and dengue by affecting lysosomal acidification, plasma membrane, and preventing virus release from lysosomes into the cytoplasm and intracellular trafficking [31,32]. Moreover, Tet entered lysosomes rapidly and triggered cholesterol accumulation (Fig. 5a, Fig. S7b–d), consistent with studies showing its selective distribution in lysosome-rich cells and tissues [26].

The NPC1-STING interaction axis provides a unique therapeutic target for blocking STING degradation in order to boost and extend innate antiviral immunity [16,33]. However, small molecules targeting this interface against viral infections have never been reported. We found that Tet binds to NPC1, preventing the selective degradation of STING in lysosomes. This prolongs antiviral response and increases IFN production, which then causes cholesterol to accumulate in lysosomes, further suppressing STING degradation and creating a feedback loop that boosts antiviral immunity via immunometabolism.

In summary, Tet emerged as the most effective small molecule from the BBAs screening. It binds to NPC1, inducing lysosomal cholesterol accumulation to resist viral entry, blocking STING lysosomal degradation and boosting innate antiviral immunity. Notably, Tet interferes with early events and viral full-lifecycle via cell cholesterol metabolism and antiviral immunity, explaining BBAs’ broad-spectrum antiviral activity. Our study highlights the importance of specific small molecules to be developed as new antiviral therapeutics via pharmacologically targeting cholesterol-mediated immunometabolism.

## MATERIALS AND METHODS

C57BL/6J mice were obtained from SPF Biotechnology Co., Ltd (Beijing, China) and housed under pathogen-free conditions. These experimental procedures adhered to the principles of animal welfare and followed the guidelines for Animals Care and Use, as approved by the Animal Ethics Committee of Beijing University of Chinese Medicine (BUCM-2023112001–4085).

Some of the data in the article were previously presented in the author's master's thesis.

Detailed descriptions of materials and methods are available in the Supplementary information.

## Supplementary Material

nwaf190_Supplemental_Files
