# Supplementary material for: Phytochemical alkaloids orchestrate immunometabolism against viral infections
Source: Natl Sci Rev. 2025 Jun 16;12(9):nwaf190. doi: 10.1093/nsr/nwaf190 (PMC12416277; doi:10.1093/nsr/nwaf190)
Supplement: nwaf190_Supplemental_Files [file nwaf190_supplemental_files.zip › NSR_MS-2024-450.R2-Supplementary Data 20250425.docx]

Supplementary Data for

**The phytochemical alkaloids orchestrate immunometabolism against viral infections**

Cuiqin Cheng ^a,^ †, Yao Wang ^b,^ †,*****, Han Wang ^c,^ †, Meiqi Zhang ^a^, Qiqi Li ^a^, Bing Xu ^c^, Lingdong Kong ^c^, Xia Liu ^a^, Yanli Yu ^c^, Yuting He ^c^, Yingjie Chu ^a^, Zhe Liu ^c^, Yuanyuan Qiao ^c^, Xinxin Yuan ^c^, Xin Jia ^c,^ *****, Anlong Xu ^a,^ *****

*** Corresponding authors.**

E-mail addresses: [yaowang@bucm.edu.cn](mailto:yaowang@bucm.edu.cn) (Yao Wang); [jiaxin@bucm.edu.cn](mailto:jiaxin@bucm.edu.cn) (Xin Jia); [xuanlong@bucm.edu.cn](mailto:xuanlong@bucm.edu.cn) (An-long Xu).

**This file includes:**

Materials and methods

Figure S1 to S7

References for Supplementary Data

**Materials and methods**

Mice

C57BL/6J mice were obtained from SPF Biotechnology Co., Ltd (Beijing, China) and housed under pathogen-free conditions. These experimental procedures adhered to the principles of animal welfare and followed the guidelines for Animals Care and Use, as approved by the Animal Ethics Committee of Beijing University of Chinese Medicine (BUCM-2023112001-4085).

Cells and viruses

The A549, L929 and THP-1 cell lines were purchased from ATCC, the Aml12 cell line was a gift from Dr. Kunpeng Liu, the *Ifnar1*^−/−^ A549 cells were constructed previously [[1](#_ENREF_1)], the MEF and PBMC cell lines were also isolated as described previously [[2](#_ENREF_2)]. The A549, MEF and L929 cell lines were cultured in Dulbecco’s modified Eagle medium (DMEM, Gibco) supplemented with 10% fetal bovine serum (FBS, Gibco) and 1% penicillin-streptomycin (PS, Gibco). Aml12 cell line needs ITS (Cyagen) additionally, and other culture conditions are the same as A549. PBMC and THP-1 cell lines were cultured in RPMI-1640 medium (Gibco) with 10% FBS and 1% PS. All cells were maintained at 37°C under 5% CO_2_.

The following viruses were obtained from our laboratory: Vesicular stomatitis virus (VSV), VSV-eGFP (enhanced green protein), encephalomyocarditis virus (EMCV), herpes simplex virus 1 (HSV-1) and human influenza virus A/Puerto Rico/8/34 (H1N1) (PR8).

Reagents and antibodies

Tetrandrine (PS0185-0020), Fangchinoline (PU0820-0025) and Cepharanthine (PS0303-0020) were purchased from Push Bio-Technology (Chengdu, China); Berbamine (B21376) and Itraconazole (B25558) were purchased from Shanghai Yuanye Bio-Technology; U18666A (T17190), Methyl-β-cyclodextrin (T4072), Cholesterol (T0760), Posaconazole (T6211), Imipramine (T20025) and DMXAA (T6273) were purchased from Topscience Co. Ltd. (Shanghai, China). ISD (interferon stimulatory DNA) was synthesized from Sangon Biotech (Shanghai, China). Cell Counting Kit-8 was purchased from Dojindo Laboratories (Kumamoto, Japan). The TRIzol Reagent and Lipofectamine™ RNAiMAX were purchased from InvivoGen (CA, USA). 2 × SYBR Green Premix (MKG787-02) was purchased from Shenzhen Meikes Biotechnology and Evo M-MLV Reverse Transcription Kit II was purchased from Hunan Ikerui Bioengineering. LysoTracker™ Deep Red (L12492) was purchased from Invitrogen. Filipin complex (F9765) was purchased from Sigma–Aldrich and Hoechst 33342 (A3472) were purchased from Apexbio Technology (Houston, TX, USA). Tissue Total Cholesterol（TC）Content Assay Kit (E1015) and Tissue Free Cholesterol（FC）Content Assay Kit (E1016) were purchased from Applygen Technologies Inc. All siRNAs were purchased from Ribobio Co., Ltd (Guangzhou, China). The antibodies were used as follows: Recombinant anti-VSV-G tag antibody (Abcam, ab183497), Anti-H1N1 Influenza A virus Nucleocapsid protein antibody (Abcam, ab104870), Recombinant Anti-Niemann Pick C1 antibody (Abcam, ab134113), Goat Anti-Rabbit IgG H&L (Alexa Fluor® 488) (Abcam, ab150077), Goat Anti-Rabbit IgG H&L (Alexa Fluor® 594) (Abcam, ab150080), Goat anti-mouse IgG H&L (Alexa Fluor® 647) pre-adsorbed secondary antibody (Abcam, ab150119), Recombinant Anti-CD63 antibody [MX-49.129.5] (Abcam, ab193349), TPCN1 Polyclonal antibody (Proteintech, 23758-1-AP), STAT1 Polyclonal antibody (Proteintech, 10144-2-AP), STAT2 Polyclonal antibody (Proteintech, 16674-1-AP), GAPDH Polyclonal antibody (Proteintech, 10494-1-AP), IRF3 Polyclonal antibody (Proteintech, 11312-1-AP), Phospho-TBK1/NAK (Ser172) (D52C2) XP® Rabbit mAb (CST 5483), TBK1/NAK (D1B4) Rabbit mAb (CST 3504), Phospho-Stat1 (Tyr701) (58D6) Rabbit mAb(CST 9167), Phospho-Stat2 (Tyr690) (D3P2P) Rabbit mAb (CST 88410), LAMP1 (D2D11) XP® Rabbit mAb (CST 9091) , ERGIC-53 antibody (B-9)(Santa cruz, sc-271517), FITC Conjugated Streptavidin (Solarbio, SF068), Anti-P4HB Mouse mAb (Hangzhou Jingjie Biotechnology, PTM-5314) and Goat Anti-Rabbit Mouse IgG-HRP (Abmart, M21003L).

Cell viability assay

A549 and L929 cells were seeded in 96-well plates at the indicated density and incubated overnight. After treatment with DMSO or different concentrations of Tet for 24 h, the cells were subsequently incubated with 10 μL cell counting kit 8-kit (CCK-8) solution for 30 minutes at 37°C. The viability of cells was analyzed by measuring the absorbance of the solution at a wavelength of 450 nm by the manufacturer's instructions.

Time-of-drug-addition assay

The time-of-drug-addition assay was performed to examine the specific stage of the viral lifecycle Tetrandrine interferes. In brief, A549 cells were infected with viruses, such as VSV-eGFP, H1N1, and EMCV, and then incubated for the indicated time at 37°C. Treatment with Tetrandrine was carried out at different stages: pre-treatment, co-treatment and post-treatment. For the pre-treatment assay, A549 cells were infected with the virus for the indicated time after the treatment with Tetrandrine for 12 h. For the co-treatment assay, A549 cells were infected with the virus and treated with Tetrandrine for the indicated time. For the post-treatment assay, A549 cells were infected with the virus and then treated with Tetrandrine for the indicated time. Flow cytometry and qRT-PCR were used to detect viral replications.

Viral attachment, entry, and post-entry assay

A549 cells were infected with VSV-eGFP, H1N1 and EMCV, and the viruses attached to the surface of cells at 4°C for 2 h. The supernatant was then removed and washed with PBS. For the viral attachment assay, the viruses were allowed to adsorb at 4°C for 2 h while incubating with Tetrandrine, and viral replication was detected by qRT-PCR. For the viral entry assay, the viruses were allowed to attach at 4°C for 2 h before treating with Tetrandrine for 1.5 h at 37°C, and qRT-PCR and flow cytometry were used to detect viral replication. For imaging of viruses, VSV-eGFP (4000 MOI) was absorbed at 4°C for 2 h, then A549 cells were washed with PBS and incubated with Tetrandrine at 37°C for 1.5 h. The cells were then fixed with 4% paraformaldehyde (PFA), permeabilized with 0.5% Triton X-100 and blocked in 5% BSA. Then, viruses were visualized using anti-GFP primary antibodies and fluorescent secondary antibodies (Alexa-488). For viral post-entry assay, viruses were allowed to adsorb at 4°C for 2 h, the supernatant was then removed and washed with PBS, and then cells were shifted to 37°C for 1.5 h, after which cells were replaced with the fresh medium containing Tetrandrine for the indicated time at 37°C, and viral replication was detected by qRT-PCR or flow cytometry.

Analogue stimulation

Polyethyleneimine (PEI) (Invitrogen, 13778150) was used for the transfection of ISD (3 μg/mL, Sangon Biotech) into THP-1 cells according to the manufacturer’s instructions, and then treated with Tetrandrine for 12 h. For DMXAA stimulation, MEF cells were stimulated with DMXAA (50 μg/mL) for 2 h followed by Tetrandrine treatment for 10 h; Aml12 cells were treated with Tetrandrine for 12 h followed by stimulation with DMXAA (50 μg/mL) for 6 h; iBMDM cells were treated with Tetrandrine for 3 h and then co-incubated with DMXAA (15 μM) for 0, 2, 4, 6, 8, 10 h.

RNA interference

RNAiMAX (Invitrogen) was used for the transfection of siRNAs into HepG2 cells following the manufacturer’s instructions. These human RNA oligos were synthesized at Guangzhou Ribobio. The sequences of siRNAs are listed in Supplementary Table 2.

Cholesterol concentration analysis

A549 cells were treated with Tetrandrine for 24 h. Total cholesterol and free cholesterol were measured with Tissue Total Cholesterol (TC) Content Assay Kit (E1015) and Tissue Free Cholesterol (FC) Content Assay Kit (E1016) following the manufacturer’s instructions.

Transcriptome analysis

A549 cells were treated with Tet, Fan, Cep, Bbm or DMSO for 24h and total RNAs were collected. Gene expression was analyzed using RNA-Seq technology. Sequencing libraries were constructed and sequenced by Beijing Biomarker Biotechnology Co (BMK) using BMKCloud (www.biocloud.net), averagely generating 5.44 GB clean data after filtering data containing adapter, ploy-N and low quality from raw data. Clean data were mapped to reference sequences using HISAT2 [[1](#_ENREF_1), [3](#_ENREF_3)]. The DEGs in our samples were obtained with a false discovery rate (FDR) < 0.05 and Log2 (fold change) > 1. Gene ontology (GO) and Kyoto Encyclopedia of Genes and Genomes (KEGG) pathway analysis was performed on Metascape (https://metascape.org). Gene set enrichment analysis (GSEA) was conducted using the GSEA software, with gene obtained from MSigDB

RNA isolation and qRT-PCR

Total RNAs were extracted using TRIzol reagent (Invitrogen). The complementary DNA template was generated by reverse transcription using Evo M-MLV Reverse Transcription Kit II (Hunan Ikerui Bioengineering). CFX96 Real-Time PCR Detection System was used for quantitative real-time PCR analysis with 2 × SYBR Green Premix (Shenzhen Meikes Biotechnology). The 2−ΔΔCt method was used to calculate relative expression changes of genes. The primer sequences are given in Supplementary Table 3.

Immunoblotting assay

Cells were lysed in lysis buffer and the protein concentrations were determined using the BCA Protein Assay Kit. Subsequently, the protein samples were separated by SDS-PAGE electrophoresis and transferred to nitrocellulose (NC) membranes, and blocked in 5% skim milk for 1 h at room temperature. Following an overnight incubation at 4°C with specific primary antibodies, the membranes were exposed to the appropriate HPR-conjugated antibodies and visualized using enhanced chemiluminescence antibodies, the membranes were exposed to the appropriate HPR-conjugated antibodies and visualized using enhanced chemiluminescence.

Flow cytometry

For drug treatment or gene knockdown, A549 cells were infected with VSV-eGFP (0.01 MOI) for 12 h. Following this, the cells were washed with PBS, treated with trypsin for digestion, and finally suspended in DMEM supplemented with 10% FBS. A total of 10,000 cells were quantified and examined on a CytoFLEX flow cytometer (Beckman Coulter) for each experimental condition. For filipin staining, cells were stained with 50 μg/mL filipin solution for 30 min at room temperature with protecting from light, and then washed with PBS. Cells were then analyzed by flow cytometer in BUV496 channel.

Enzyme-linked immunosorbent assay (ELISA)

Cell supernatant and mouse serum were collected for ELISA. The concentration of IFN-β was measured using ELISA kits from R&D Systems according to the manufacturer’s instructions. The concentrations of IL-6 and TNF-α were determined by utilizing ELISA Kits provided by Proteintech.

Immunoprecipitation

A549 and HepG2 cells (3 × 10^7^) were lysed in cell lysis buffer (Beyotime Biotechnology) containing phosphatase inhibitor cocktail and protease inhibitor cocktail. Then, the cell lysates were incubated with immobilized Bio-Tet, Bio-Tet and Tet, or NC at 4°C overnight, followed by incubation with 25 µL of streptavidin magnetic beads (Thermo Fisher) for another 6 h. After adequately washing, the immunoprecipitates were eluted by heating to 100°C in cell lysis buffer, and immunoblotting was used to analyze the target proteins.

Confocal microscopy

Cells were fixed with 4% PFA for 20 min, permeabilized with 0.2% Triton X-100 for 20 min, and blocked in 5% BAS for 1 h. After incubating with primary antibodies at 4℃ overnight, fluorescent secondary antibodies (Alexa-488, Alexa-594, Alexa-647) were added at RT for 1 h. Confocal microscopy analyses were carried out using Olympus FV3000 confocal microscope, and Leica TCS SP8 confocal microscope. For Filipin staining, 5 mg/mL Filipin reserve solution dissolved in ethanol was diluted to 200 μg/mL using PBS containing 10% fetal bovine serum; cells were then stained with this diluted Filipin solution for 2 h at room temperature while being protected from light.; and then washed with PBS. Cells were then analyzed for Filipin staining signals by microscopy in the DAPI channel. For Lysotracker staining, cells were stained with 75nM Lysotracker solution for 30 min at room temperature with protection from light; and then washed with PBS. Cells were then analyzed for signals by microscopy in the AF647 channel.

Molecular docking

The NPC1 crystal structure (PDB ID:6W5T) was obtained from the Protein Data Bank database. The 3D structure of Tetrandrine, Fangchinoline, Cepharanthine, Berbamine, Itraconazole and U18666A were downloaded from the Pubchem database. AutoDockVina1.1.2 and PyMOL2.3.0 were used to perform the docking simulation and analyze docking results.

Cellular thermal shift assay (CETSA)

The A549 and HepG2 cells were frozen and thawed three times in liquid nitrogen to lyse them. The lysed cells were then centrifuged at 4°C at 15,000 × g for 40 minutes to obtain the total protein. Next, the proteins were treated with either DMSO or Tetrandrine (150 μM) at room temperature for 1 hour. The protein mixture was then divided into PCR tubes and heated at various temperatures (37, 41, 44, 47, 50, 53, 56, 59, 63, and 67°C) for 3 min, followed by cooling on ice and centrifugation at 4°C at 15,000 × g for 40 minutes. The resulting supernatant was used for an immunoblot assay.

Drug affinity responsive target stability (DARTS)

The total protein of A549 cells was obtained and diluted with the TNC buffer, which consists of 50 mM Tris-HCl (pH 8.0), 50 mM NaCl and 10 mM CaCl2. The concentration of the protein was determined and adjusted to 5 µg/µL. Following this, the total protein was incubated with either DMSO or Tetrandrine (150 µM) at room temperature for 1 hour. Afterward, the mixture was evenly divided and subjected to pronase digestion for 30 minutes at room temperature. The pronase reaction was halted by adding 5 × SDS loading buffer, and the resulting digestion fragments were analyzed using an immunoblot assay.

Lysosome immunoprecipitation followed by density gradient ultracentrifugation (LysoIP-DEG)

The LysoIP-DEG was performed as previously described with minor modifications [[4](#_ENREF_4)]. HepG2 cells were seeded on 10 cm cell culture plates and transfected with TMEM192-3×Flag plasmid. Cells were then washed with cold PBS, scraped, centrifuged at 300 × g for 5 min at 4 °C, and resuspended with LysoIP buffer (140 mM KCl, 5 mM MgCl2, 50 mM sucrose, 20 mM HEPES pH 7.4 supplemented with protease inhibitors). Cells were gently homogenized using a 26 G 1/2 needle attached to a 1 ml syringe, with cells being passed through the needle 20 times. The cell lysate was then centrifuged at 2,000 × g for 10 min at 4 °C, leading to the retrieval of the supernatant enriched with cellular organelles such as lysosomes. 50 μL supernatant was reserved for input, while the remaining volume was carefully transferred to the microtub containing 40 μl of ANTI-FLAG M2 Affinity Gel (Sigma, A2220). After overnight incubation with gentle agitation, the beads were washed four times with LysoIP buffer and eluted by 800 μL lysis buffer including 3×FLAG peptide for LysoIP-DEG (1% (v/v). The eluted lysosomes were mixed with an equal volume of 80% sucrose within a denaturation buffer devoid of detergents and then transferred to ultracentrifuge tubes. A non-continuous sucrose gradient ranging from 35% to 5% was carefully layered onto the specimen and subsequently subjected to ultracentrifugation at a force of 200,000g for a duration of 16 h. Following the centrifugal process, 11 distinct fractions were retrieved in a top-to-bottom manner and subsequently treated using precipitation techniques. The collected samples were dissolved in 50 µL 2 × SDS loading buffer and then assessed through western blotting analysis.

Knockout of *TPCN1* and *TPCN2* by the CRISPR-Cas9 system

*TPCN1*^−/−^, *TPCN2*^−/−^A549 cells were constructed using the CRISPR-Cas9 gene-editing system. Small guide RNAs (sgRNAs) targeting the genome sequence of *TPCN1* or *TPCN2* genes were ligated into lenti-CRISPR-Cas9 vectors. The CRISPR plasmids were infected into cells using a lentivirus. Single-cell clones of the infected cells were then selected and validated by immunoblotting. The sgRNA sequences were designed using an online tool as follows:

Human *TPCN1*-sgRNA-1: Forward: 5’- CACCGCCACTTCAGCCACTGCCACC -3’, reverse: 5’- AAACGGTGGCAGTGGCTGAAGTGGC -3’;

Human *TPCN1*-sgRNA-2: Forward: 5’- CACCGCCTGGTGGCAGTGGCTGAAG -3’, reverse: 5’- AAACCTTCAGCCACTGCCACCAGGC -3’;

Human *TPCN1*-sgRNA-3: Forward: 5’- CACCGTGAAGTGGCGGCGGCTTCGG -3’, reverse: 5’- AAACCCGAAGCCGCCGCCACTTCAC -3’;

Human *TPCN2*-sgRNA-1: Forward: 5’- CACCGCGCGTGGGCTGCTGGATGG -3’, reverse: 5’- AAACCGCGCACCCGACGACCTACC -3’;

Human *TPCN2*-sgRNA-2: Forward: 5’- CACCGAGGGGCTCCGACTCCGCCTG-3’, reverse: 5’- AAACCAGGCGGAGTCGGAGCCCCTC -3’;

Human *TPCN2*-sgRNA-3: Forward: 5’- CACCGCTGGATGGCGGAACCCCAGG -3’, reverse: 5’- AAACCCTGGGGTTCCGCCATCCAGC -3’.

Knockdown of *NPC1* by the CRISPR interference (CRISPRi) system

The dCAS9-KRAB vector and sgRNA were used to construct a dCas9-sgRNA complex targeting NPC1 following a standard protocol of CRISPRi [[5](#_ENREF_5)]. The sgRNA was synthesized and annealed before being ligated into the dCAS9-KRAB vector. The dCas9-sgRNA complex was packaged and infected into cells using a lentivirus. Single-cell clones of the infected cells were then selected and subjected to immunoblotting to measure the efficacy of gene knockdown. The sgRNA sequences were designed using an online tool as follows:

GFP-sgRNA (as control): Forward: 5’- CACCGGGGCGAGGAGCTGTTCACCG -3’, reverse: 5’- AAACCGGTGAACAGCTCCTCGCCCC -3’;

Mouse *NPC1*-sgRNA-1: Forward: 5’- CACCGTCGGAACCGGCGCCTGACCA-3’, reverse: 5’- AAACTGGTCAGGCGCCGGTTCCGAC -3’;

Mouse *NPC1*-sgRNA-2: Forward: 5’- CACCGCAGGAGCAGGCGCTGACCG -3’, reverse: 5’- AAACCGGTCAGCGCCTGCTCCTGC -3’.

*In vivo* experiments

For *in vivo* VSV infection, 6-8 weeks C57/BL6J mice were infected with VSV at a dosage of 2 × 10^8^ PFU per mouse. This was done through a 2-day intragastric gavage (i.g) treatment of Tetrandrine tablets (10, 30, 60 mg/kg^-1^/day^-1^) from Zhejiang Jinhua Kangenbei Biopharmaceutical Co., Ltd., or a 1-day intraperitoneal injection of Tetrandrine (10, 30, 60 mg/kg^-1^/day^-1^) from Jiangxi Yintao Pharmaceutical Co., Ltd., or a 0.5-day treatment of DMXAA (10 mg/kg^-1^/day^-1^) through intraperitoneal injection. Mouse serum, lungs, spleens, and livers were collected 24 h after infection to measure cytokine levels and viral loads using ELISA, immunoblotting, and qRT-PCR. The mouse lungs were preserved in a 4% paraformaldehyde solution for Hematoxylin and eosin (H&E) staining.

For the *in vivo* H1N1 infection experiment, 6-8 weeks C57/BL6J mice were infected with H1N1 (1 × 10^4^ PFU per mouse) by direct delivery to the nares. Before the infection, the mice were treated with Tetrandrine injection (15 mg/kg^-1^/day^-1^) by intranasal administration for 1 day, or Oseltamivir (40 mg/kg^-1^/day^-1^) by intragastric gavage for 1 day. The survival of the infected mice was monitored for a period of 10 days. Bronchoalveolar lavage fluid and lungs were collected from the mice after 4 days of infection to determine the viral load using qRT-PCR. Mice lungs and bronchus were fixed in a 4 % paraformaldehyde solution for H&E staining and Immunohistochemistry (IHC).

For H&E staining, mice lung tissues fixed in 4 % paraformaldehyde for 12 h were dewaxed with xylenes and rehydrated with a gradient of alcohol, then stained with hematoxylin for 10 min, eosin for 3 min. After sealing with neutral gum, the lung tissues were observed using a super-resolution automatic scanning microscope.

For IHC experiments, mice tissues fixed in 4 % paraformaldehyde were embedded in paraffin and cut into 3-mm-thick sections. To visualize replicated H1N1, anti-influenza a virus nucleoprotein antibody (Abcam, ab20343) was used as a primary antibody after antigen retrieval. After performed with a secondary antibody, immune complexes were visualized by treated with 3,3’-diaminobenzidine tetrahydrochloride staining.

Statistics and reproducibility

The experimental data were presented as mean ± SD and analysed using GraphPad Prism. Statistical significance for two groups was determined using an unpaired two-tailed Student's *t*-test, while three or more groups were analyzed using a one-way ANOVA with a Bonferroni post-test. A significance level of *P* < 0.05 was considered statistically significant.

**Supplementary Figure Legends**

**Figure S1. Loss of NPC1 confers resistance to viral infection. a** qRT-PCR analysis of the expression of cholesterol transport-related genes in HepG2 cells transfected with siRNAs for 72 h.**b** Cell viability analysis of HepG2 cells transfected with siRNAs for 72 h. **c** qRT-PCR analysis of VSV mRNA expression in HepG2 cells transfected with indicated siRNAs for 72 h following infection with VSV for 12 h. **d** Immunoblotting of NPC1 expression in sgRNA-EV, *Npc1*#1, and *Npc1*#2 cells. **e** Indicated gene expression in the cerebellum of WT and *Npc1*^-/-^ mice (GSE20450). For a, b, c, n = 3 independent experiments. All data are presented as mean ± SD. *P* values were calculated using unpaired two-tailed Student’s *t-*test (a) or one-way ANOVA (b). NS, not significant.

**Figure S2. Bis-benzylisoquinoline alkaloids inhibit the replication of H1N1, EMCV, and HSV-1 *in vitro*.** **a-c** L929 cells were treated with increasing concentrations of the indicated compounds (Tet, Fan, Cep, Bbm) and infected with H1N1 (a), EMCV (b), and HSV-1 (c) in the presence of the compounds for the indicated duration. Dose-response curves for infectivity (red) and cell viability (blue) were generated, with the data normalized to the average of DMSO-treated wells. **d** Confocal microscopy analysis of NP (red) protein of H1N1and DAPI (blue) in A549 cells treated with DMSO or Tet followed by H1N1 infection for 12 h, scar bar = 50 μm. **e** Immunoblotting of NP protein of H1N1 in A549 cells co-incubated with Tet and H1N1 for 16 h. For a, b, c, d, n = 3 independent experiments. All data are presented as mean ± SD. *P* values were calculated using one-way ANOVA.

**Figure S3. Tet interferes with the entry and post-entry steps of the virus life cycle.** **a** Schematic diagram illustrating the time-of-drug-addition assay of Tet, including pre-treatment, co-treatment, and post-treatment. **b** Flow cytometry analysis of the percentage of eGFP-positive cells in VSV-eGFP infected A549 cells with Tet (2.5, 5, 10 μM) pre-treatment, co-treatment, or post-treatment. **c-d** qRT-PCR analysis of H1N1 (c) or EMCV (d) mRNA expression in A549 cells infected with the indicated virus and treated with Tet during pre-treatment, co-treatment, and post-treatment. **f, g** Flow cytometry analysis of A549 cells challenged by VSV-eGFP with Tet incubation during VSV-eGFP entry stage (f) or post-entry stage (g). **h-j** H1N1 mRNA expression in A549 cells treated with Tet during H1N1 attachment stage (h), entry stage (i), or post-entry stage (j). **k-m** EMCV mRNA expression in A549 cells treated with Tet during EMCV attachment stage (k), entry stage (l), and post-entry stage (m). For b-m, n = 3 independent experiments. All data are presented as mean ± SD. *P* values were calculated using one-way ANOVA. NS, not significant.

**Figure S4. Tet inhibits virus replication independent of Two-pore channels (TPCs).** **a** Immunoblotting of TPCN1 in WT and *TPCN1*^-/-^ A549 cells. **b** Flow cytometry analysis of the percentage of eGFP-positive cells in WT, *TPCN1*^-/-^, and *TPCN2*^-/-^A549 cells infected with VSV-eGFP following treatment of Tet. **c** qRT-PCR analysis of H1N1 mRNA expression in WT, *TPCN1*^-/-^, and *TPCN2*^-/-^ A549 cells infected with H1N1 following treatment of Tet. **d** Flow cytometry analysis of A549 cells with normal Ca^2+^ or Ca^2+^ free medium followed by VSV-eGFP infection with or without Tet. **e** Statistical analysis of the data from the multiple repeated experiments in d. **f** qRT-PCR analysis of H1N1 mRNA expression in A549 cells following treatment of Tet, nimodipine, or combination of Tet and nimodipine. For b, c, e, f, n = 3 independent experiments. All data are presented as mean ± SD. *P* values were calculated using one-way ANOVA.

**Figure S5. Tet activates STING-dependent antiviral innate immune responses.** **a** Immunoblotting of phosphorylated TBK1 (p-TBK1), TBK1, phosphorylated STAT1 (p-STAT1), and STAT1 in MEF cells treated with 10 μM Tet for indicated times. **b** Immunoblotting of p-TBK1, TBK1, p-STAT1, STAT1 in THP-1 cells treated with Tet for 24 h. **c** qRT-PCR analysis of *Ifnb1, Ifit1, Ifit2, and Ifi44* mRNA expression in mouse lungs after intraperitoneal injections of Tet (0, 30, 60 mg kg^-1^ d^-1^) for 48 h. **d-f** qRT-PCR analysis of *Ifnb1, Ifit1, Ifit2,* and *Ifi44* mRNA expression in mouse livers (e), lungs (f), spleens (g) after intraperitoneal injections of Tet (60 mg kg^-1^ d^-1^) for 0, 24, 48 h. **g** The mRNA levels of *Sting* were measured in WT or *Sting*^-/-^ MEF. **h** qRT-PCR analysis of *Ifi44, Ccl5,* and *Cxcl10* mRNA expression in WT or *Sting*^-/-^ MEF cells stimulated with DMXAA (50 μg‧ml^−1^) for 2 h and followed by 10 μM Tet for 6 h. **i** Immunoblotting of IFNAR1 in WT or *IFNAR1*^-/-^ A549 cells. **j** qRT-PCR analysis of *IFIT1* and *IFI44* mRNA expression in WT or *IFNAR1*^-/-^ A549 cells treated with 10 μM Tet for 24 h. **k** Confocal microscopy analysis of lysotracker (red) and cholesterol-staining agent filipin (green) in A549 cells treated with 10 μM Tet, 10 μM U18666A or 1 μM IFN-β for 24 h. scale bar: 10 μm. For c, d, e, f, g, h, j, k, n = 3 independent experiments. All data are presented as mean ± SD. *P* values were calculated using unpaired two-tailed Student’s *t-*test. NS, not significant.

**Figure S6. Bis-benzylisoquinoline alkaloids modulate cholesterol metabolism and activate antiviral innate immunity. a-d** GO enrichment analysis of upregulated genes in A549 cells treated with 10 μM Tet, Fan, Cep or Bbm for 24 h. **e** GSEA analysis of upregulated genes in A549 cells treated with Fan, Cep or Bbm. **f** Heatmap of cholesterol metabolism genes and ISGs in WT or *IFNAR1*^-/-^ A549 cells treated with Tet, Fan, Cep, or Bbm. **g** KEGG pathway and Wikipathways analysis of upregulated genes in A549 cells treated with Tet, Fan, Cep or Bbm.

**Figure S7. Tet coordinate the dual functions of inhibiting viral entry and activating antiviral innate immunity through NPC1/STING axis.** **a** Molecular structure of Bio-Tetrandrine (Bio-Tet). **b** Quantification of Bio-Tet and CD63 co-localization from the experiment in (Figure 5a). n= 15 cells/condition. **c** Confocal microscopy analysis of the co-localization of Bio-Tet (green) and lysotracker (red) in A549 cells treated with Bio-Tet for indicated times. Scale bar: 10 μm. **d** Quantification of Bio-Tet and lysotracker co-localization from the experiment in (c). n = 15 cells/condition. **e** Flow cytometry analysis of the percentage of eGFP-positive cells in A549 cells infected with VSV-eGFP following treatment of Bio-Tet. **f** qRT-PCR analysis of cholesterol metabolism genes and ISGs expression in A549 cells treated with Bio-Tet for 24 h. **g** Confocal microscopy analysis of LAMP1 (red) and filipin (green) in HepG2 cells treated with si *NPC1* for 72 h or 10 μM Tet for 24 h. scale bar: 10 μm. **h** Quantification of Bio-Tet and NPC1 co-localization from the experiment in (Figure 5d). n= 15 cells/condition. **i** Docking model showing the interaction between U18666A, Itraconazole, Tetrandrine, Fangchinoline, Cepharanthine, Berbamine and NPC1 protein. **j** CETSA analysis of NPC1 with Tet in A549 cells. **k** qRT-PCR analysis of *NPC1* mRNA expression in HepG2 cells transfected with si*NPC1* for 72 h. **l** Immunoblotting of NPC1 expression in HepG2 cells transfected with siCon or siNPC1 for 72 h. **m** Immunoblotting of NPC1 expression in sgRNA-EV cells, sgRNA *Npc1* cells, and sgRNA *Npc1* cells transfected with Flag-NPC1. **n** qRT-PCR analysis of mRNA expression (left panel: H1N1, right panel: EMCV) in A549 cells infected with indicated virus treated with 10 μM Tet or 10 μM U18666A in different administration methods. **o** Immunoblotting of STING, p-TBK1, or p-STAT1 expression levels in iBMDM cells pretreated with DMSO or Tet (10 μM) for 3 h or BafA1 (100 nM) for 8 h and then co-incubated with DMXAA (15 μM) for indicated times. **p** Evaluation of immunopurified lysosomes in Figure 5j. Cell lysates and immunoprecipitated samples were analyzed using TMEM192-3×FLAG as a bait protein for rapid isolation of lysosomes. For b, c, d, e, f, g, h, k, n, n = 3 independent experiments. All data are presented as mean ± SD. *P* values were calculated using unpaired two-tailed Student’s *t-*test (b, d, f, h, k) or one-way ANOVA (n). NS, not significant.

**Reference:**

1. Wang Y, Yuan S, Jia X *et al.* Mitochondria-localised ZNFX1 functions as a dsRNA sensor to initiate antiviral responses through MAVS. *Nat Cell Biol* 2019; **21**: 1346-1356.

2. Jia X, Yuan S, Wang Y *et al.* The role of alternative polyadenylation in the antiviral innate immune response. *Nat Commun* 2017; **8**: 14605.

3. Kim D, Langmead B and Salzberg SL. HISAT: a fast spliced aligner with low memory requirements. *Nat Methods* 2015; **12**: 357-360.

4. Roh K, Noh J, Kim Y *et al.* Lysosomal control of senescence and inflammation through cholesterol partitioning. *Nature Metabolism* 2023; **5**: 398-+.

5. Larson MH, Gilbert LA, Wang X *et al.* CRISPR interference (CRISPRi) for sequence-specific control of gene expression. *Nat Protoc* 2013; **8**: 2180-2196.
