# Supplementary material for: Phytochemical alkaloids orchestrate immunometabolism against viral infections
Source: Natl Sci Rev. 2025 Jun 16;12(9):nwaf190. doi: 10.1093/nsr/nwaf190 (PMC12416277; doi:10.1093/nsr/nwaf190)
Supplement: nwaf190_Supplemental_Files [file nwaf190_supplemental_files.zip › NSR_MS-2024-450.R2-Supplementary Tables 20250425.docx]

Supplementary Data for

**The phytochemical alkaloids orchestrate immunometabolism against viral infections**

Cuiqin Cheng ^a,^ †, Yao Wang ^b,^ †,*****, Han Wang ^c,^ †, Meiqi Zhang ^a^, Qiqi Li ^a^, Bing Xu ^c^, Lingdong Kong ^c^, Xia Liu ^a^, Yanli Yu ^c^, Yuting He ^c^, Yingjie Chu ^a^, Zhe Liu ^c^, Yuanyuan Qiao ^c^, Xinxin Yuan ^c^, Xin Jia ^c,^ *****, Anlong Xu ^a,^ *****

*** Corresponding authors.**

E-mail addresses: [yaowang@bucm.edu.cn](mailto:yaowang@bucm.edu.cn) (Yao Wang); [jiaxin@bucm.edu.cn](mailto:jiaxin@bucm.edu.cn) (Xin Jia); [xuanlong@bucm.edu.cn](mailto:xuanlong@bucm.edu.cn) (An-long Xu).

**This file includes:**

Supplementary Table 1 to 3

**Supplementary Tables**

**Supplementary Table 1: 619 differentially expressed genes in sgRNA NPC1 cells compared to sgRNA EV cells.**

| **Gene Symbol** | **log2 (sgRNA NPC1 vs EV)** | **FDR (sgRNA NPC1 vs EV)** |  |
| --- | --- | --- | --- |
| Rsph3b | 1.290014291 | 1.85E-09 | up |
| Gm10058 | 10.34429591 | 4.15E-04 | up |
| Gm2832 | 1.817005092 | 2.97E-04 | up |
| 1700049E17Rik2 | 1.503452222 | 9.69E-04 | up |
| R3hdml | 2.132595751 | 1.59E-89 | up |
| Pcsk9 | 1.722154461 | 1.48E-245 | up |
| Klhl3 | 1.108524457 | 5.40E-04 | up |
| Zfp534 | 11.26150731 | 2.78E-07 | up |
| Gm21154 | 1.756060964 | 5.49E-05 | up |
| Gm21304 | 14.05942896 | 4.82E-51 | up |
| Zfp990 | 2.252255361 | 1.63E-06 | up |
| Cyp4v3 | 1.925999419 | 1.45E-06 | up |
| Mapkapk3 | 2.847996907 | 1.75E-06 | up |
| Gm35588 | 5.426264755 | 9.07E-23 | up |
| Gm36718 | 2 | 9.09E-04 | up |
| Radx | 11.28771238 | 1.42E-07 | up |
| Gstt3 | 4.392317423 | 3.07E-10 | up |
| Chchd10 | 2.024166311 | 2.04E-33 | up |
| Synpo | 3.584962501 | 2.14E-05 | up |
| Cyp27a1 | 4.087462841 | 2.60E-04 | up |
| Gm42346 | 1.123539034 | 3.90E-16 | up |
| Gpsm3 | 2.722466024 | 9.05E-06 | up |
| Arap3 | 2.807354922 | 3.04E-06 | up |
| Aldh1l1 | 2.987927168 | 4.82E-41 | up |
| Ankrd1 | 1.252520184 | 7.63E-160 | up |
| Olr1 | 4.523561956 | 6.31E-06 | up |
| Adamts7 | 4.285402219 | 2.07E-09 | up |
| Gm5798 | 1.732433425 | 2.96E-04 | up |
| Ston2 | 2.489584453 | 1.64E-78 | up |
| Fam72a | 1.250808104 | 3.83E-11 | up |
| F11 | 11.10328781 | 1.06E-06 | up |
| Fdps | 1.46869879 | 0.00E+00 | up |
| Krt7 | 2.670353992 | 4.37E-40 | up |
| Acat2 | 1.725025051 | 6.14E-214 | up |
| Lmx1a | 1.604071324 | 6.01E-37 | up |
| Slc18a1 | 2.273018494 | 2.21E-04 | up |
| Zfp979 | 4.690195687 | 1.34E-13 | up |
| Acp5 | 1.666955873 | 6.52E-16 | up |
| Chrna1 | 3.690541257 | 2.32E-120 | up |
| Zfp988 | 10.64295422 | 1.12E-04 | up |
| Gm52800 | 1.035743798 | 7.97E-35 | up |
| Adssl1 | 1.762500686 | 2.37E-09 | up |
| Afp | 3.917778912 | 2.82E-144 | up |
| Angpt2 | 1.050626073 | 2.46E-09 | up |
| Aldoc | 1.175086707 | 2.45E-04 | up |
| Apobec1 | 2.613817363 | 1.91E-30 | up |
| Arg1 | 4.169925001 | 1.42E-04 | up |
| LOC118567664 | 1.888499736 | 4.26E-08 | up |
| Ass1 | 7.294620749 | 1.26E-90 | up |
| Atp1b1 | 4.321928095 | 1.30E-46 | up |
| Azgp1 | 4.700439718 | 4.93E-13 | up |
| Bdkrb2 | 11.45121111 | 1.88E-08 | up |
| Btg3 | 1.57792495 | 1.84E-32 | up |
| Camk4 | 1.166009951 | 2.25E-05 | up |
| Car2 | 2.669851398 | 3.13E-11 | up |
| Casp1 | 2.807354922 | 3.05E-06 | up |
| Casp4 | 10.34429591 | 4.14E-04 | up |
| Cd37 | 2.618909833 | 4.85E-07 | up |
| Cdh16 | 1.674309986 | 2.26E-130 | up |
| Cish | 1.669851398 | 9.66E-04 | up |
| Clu | 1.01342342 | 0 | up |
| Col11a2 | 1.127111918 | 1.01E-04 | up |
| Cryab | 1.092566119 | 1.35E-16 | up |
| Csf3 | 1.618909833 | 2.93E-04 | up |
| Csn3 | 11.85330956 | 4.19E-11 | up |
| Cybb | 1.02727293 | 1.27E-11 | up |
| Cyp2a5 | 8.142957954 | 3.67E-250 | up |
| Cyp51 | 1.878222794 | 0 | up |
| Arid3a | 1.502500341 | 1.02E-05 | up |
| Dtna | 4.906890596 | 2.67E-15 | up |
| Opn3 | 1.137503524 | 5.58E-04 | up |
| Eps8 | 1.865863327 | 8.82E-154 | up |
| F2rl1 | 1.358686645 | 1.67E-30 | up |
| Fdft1 | 2.024352424 | 2.64E-239 | up |
| Fgfbp1 | 3.456267812 | 1.24E-178 | up |
| Ccn2 | 2.25919234 | 3.04E-113 | up |
| Fzd7 | 1.352301744 | 3.13E-08 | up |
| Gcnt1 | 2.365350375 | 1.50E-49 | up |
| Gda | 1.584962501 | 3.05E-19 | up |
| Ghr | 1.897497461 | 8.44E-43 | up |
| Gja1 | 1.736500134 | 1.44E-92 | up |
| Gsta3 | 1.166802959 | 4.29E-144 | up |
| H2-DMb1 | 1.95419631 | 5.40E-04 | up |
| H2-Q1 | 1.247565546 | 2.10E-06 | up |
| Hebp1 | 1.130060541 | 3.62E-17 | up |
| Hk2 | 1.170965532 | 6.82E-70 | up |
| Hmgcr | 1.238368411 | 1.20E-203 | up |
| Foxa1 | 1.070389328 | 5.50E-09 | up |
| Hsd17b7 | 1.633932476 | 1.58E-135 | up |
| Hspb1 | 3.247927513 | 1.35E-07 | up |
| Hspa2 | 1.210217707 | 5.44E-07 | up |
| Idh1 | 1.019540186 | 2.80E-99 | up |
| Ier3 | 1.156172735 | 4.78E-76 | up |
| Il15 | 2.076350886 | 1.31E-11 | up |
| Kcnab2 | 2.570165499 | 1.40E-127 | up |
| Kcnu1 | 1.468148836 | 1.34E-06 | up |
| Kif21b | 1.491853096 | 4.93E-04 | up |
| Fabp5 | 1.036815158 | 5.13E-191 | up |
| Klf12 | 11.73131903 | 3.23E-10 | up |
| Klra1 | 1.730004328 | 3.57E-05 | up |
| Kng1 | 11.45121111 | 1.88E-08 | up |
| Sspn | 3.662965013 | 2.59E-08 | up |
| Lamc2 | 1.075407403 | 1.33E-19 | up |
| Ldlr | 1.182373603 | 4.23E-166 | up |
| Lepr | 1.772589504 | 1.60E-04 | up |
| Lifr | 2.678071905 | 2.33E-10 | up |
| Lpl | 1.277704095 | 2.13E-15 | up |
| Lss | 1.625376769 | 3.33E-177 | up |
| Lyz2 | 3.169925001 | 6.95E-04 | up |
| Havcr2 | 2.397592365 | 2.48E-37 | up |
| Smad6 | 1.071790683 | 2.69E-05 | up |
| Apobr | 2.958842675 | 5.88E-53 | up |
| Maoa | 1.047412502 | 2.89E-108 | up |
| Masp1 | 1.313838777 | 1.96E-69 | up |
| Mep1a | 1.906890596 | 2.70E-08 | up |
| Mmp13 | 1.61533615 | 2.63E-12 | up |
| Map6 | 2.28757659 | 3.34E-11 | up |
| Mvk | 1.509206663 | 1.31E-42 | up |
| Naip5 | 1.382851576 | 3.15E-04 | up |
| Nsdhl | 1.932120144 | 1.93E-141 | up |
| Ddr2 | 4.832890014 | 1.91E-14 | up |
| P2rx4 | 1.489965987 | 1.07E-41 | up |
| Pea15a | 1.398121065 | 0 | up |
| Abcb1a | 1.127201831 | 7.48E-62 | up |
| Prkcg | 1.795180208 | 3.04E-06 | up |
| Pla2g5 | 3.650253961 | 1.58E-23 | up |
| Lcp1 | 3.227535725 | 5.07E-52 | up |
| Septin5 | 1.346175641 | 5.04E-08 | up |
| Mvd | 1.791524693 | 3.24E-93 | up |
| Tmlhe | 1.343231608 | 1.37E-19 | up |
| Ptprb | 5.847996907 | 1.14E-234 | up |
| Rab17 | 1.003497444 | 6.53E-17 | up |
| Cnksr1 | 3.718818247 | 7.61E-17 | up |
| Utp14b | 1.135663914 | 2.53E-19 | up |
| Zfp982 | 2.243444604 | 1.31E-11 | up |
| Renbp | 1.627582135 | 4.37E-48 | up |
| Mst1r | 1.299560282 | 2.85E-08 | up |
| Rora | 1.339486466 | 2.28E-08 | up |
| Trim30a | 4.857980995 | 9.92E-15 | up |
| S100a8 | 2.584962501 | 3.31E-04 | up |
| Scn8a | 3.222392421 | 8.01E-11 | up |
| Ccl2 | 1.797065359 | 0 | up |
| Ccl20 | 3.080064389 | 8.97E-69 | up |
| Ccl7 | 1.278716028 | 1.26E-17 | up |
| Selp | 12.34429591 | 1.46E-15 | up |
| Sema3a | 2.410019425 | 2.62E-39 | up |
| Slc4a3 | 10.45121111 | 2.15E-04 | up |
| Slfn2 | 1.590386173 | 1.44E-42 | up |
| Slpi | 3.415037499 | 9.46E-07 | up |
| Snai1 | 2.765534746 | 5.27E-06 | up |
| Snca | 1.050768611 | 3.86E-31 | up |
| Soat1 | 1.125200557 | 2.39E-19 | up |
| Sox9 | 1.133266531 | 4.49E-20 | up |
| Serpinb9b | 2.602869068 | 0 | up |
| Serpinb5 | 1.388504933 | 2.87E-141 | up |
| Serpinb8 | 1.922320636 | 1.36E-24 | up |
| Pde2a | 4.321928095 | 4.12E-05 | up |
| Sqle | 1.469837501 | 0 | up |
| Stc2 | 2.304854582 | 2.01E-11 | up |
| Hmgcs1 | 1.575418728 | 0 | up |
| Bhlhe40 | 1.18843047 | 5.40E-118 | up |
| Dhtkd1 | 1.339596743 | 5.34E-04 | up |
| Shank2 | 3.523561956 | 2.31E-96 | up |
| Atg9b | 1.031535573 | 6.94E-16 | up |
| Tcf7 | 1.125294128 | 3.31E-04 | up |
| Pak6 | 3.754887502 | 9.00E-12 | up |
| Tcn2 | 1.166009951 | 6.72E-10 | up |
| Aldh5a1 | 1.169925001 | 1.60E-07 | up |
| 4930486L24Rik | 1.78447631 | 8.24E-73 | up |
| Cped1 | 4 | 4.75E-04 | up |
| Slc35f1 | 2.786596362 | 1.49E-11 | up |
| Rnf144b | 6.845490051 | 1.09E-32 | up |
| Thbd | 11.39874369 | 3.67E-08 | up |
| 3830406C13Rik | 1.094363505 | 6.33E-15 | up |
| Ube2e2 | 1.238159737 | 8.33E-06 | up |
| Tasor | 2.677690307 | 9.34E-72 | up |
| Tspan7 | 1.179202815 | 1.95E-18 | up |
| Tnfrsf9 | 1.123058396 | 3.14E-08 | up |
| Tpd52l1 | 1.668378509 | 4.48E-38 | up |
| Upk1b | 1.870716983 | 2.02E-101 | up |
| Vnn1 | 1.010836661 | 2.35E-101 | up |
| Poglut1 | 3.645991934 | 2.01E-69 | up |
| Wnt7a | 2.007494537 | 1.83E-21 | up |
| Xlr3a | 11.10328781 | 1.06E-06 | up |
| Plaat3 | 1.209453366 | 1.07E-04 | up |
| Gstp3 | 2.772589504 | 4.12E-07 | up |
| Rab7b | 1.181838323 | 1.76E-08 | up |
| Rab29 | 1.012600037 | 2.17E-05 | up |
| Zfp37 | 1.089637212 | 1.58E-04 | up |
| Zfp57 | 1.243030931 | 5.34E-61 | up |
| Proser2 | 1.139551352 | 1.53E-07 | up |
| Gsn | 1.154246788 | 1.35E-28 | up |
| Sdcbp2 | 1.655351829 | 5.19E-06 | up |
| Slc17a9 | 1.554588852 | 2.24E-04 | up |
| Pbxip1 | 3.824428435 | 1.82E-18 | up |
| Asap3 | 1.710493383 | 6.34E-04 | up |
| Insig1 | 2.236124304 | 1.64E-279 | up |
| Oasl1 | 1.925999419 | 9.56E-30 | up |
| Mgam | 1.847996907 | 2.96E-04 | up |
| Tcaf2 | 12.03617361 | 1.39E-12 | up |
| Atp1a3 | 1.906890596 | 8.50E-04 | up |
| Mogat2 | 1.914270126 | 1.02E-05 | up |
| Cbr4 | 1.832122827 | 1.44E-45 | up |
| Spire2 | 10.34429591 | 4.14E-04 | up |
| Dmxl2 | 1.00666373 | 2.50E-09 | up |
| Mrgpra4 | 4.129283017 | 2.54E-08 | up |
| Lancl3 | 2.736965594 | 7.09E-07 | up |
| Gpr135 | 2.471922563 | 9.16E-07 | up |
| Ogdhl | 1.623851514 | 6.19E-16 | up |
| Nt5e | 1.161463423 | 1.74E-17 | up |
| Slco2a1 | 1.185253915 | 8.06E-259 | up |
| Pgap1 | 1.248297863 | 1.72E-27 | up |
| Usp18 | 3.209453366 | 2.41E-07 | up |
| Tmem130 | 2.222392421 | 4.37E-16 | up |
| Ano2 | 3 | 9.55E-05 | up |
| Ppp1r9a | 1.056583528 | 5.18E-04 | up |
| Parp12 | 3.232660757 | 3.31E-09 | up |
| Zscan4c | 1.350792912 | 1.76E-05 | up |
| Nxf3 | 6.497186541 | 4.96E-76 | up |
| Pwwp3b | 1.741466986 | 2.79E-10 | up |
| Oas1a | 11.15418521 | 1.06E-06 | up |
| Anxa10 | 3 | 1.86E-07 | up |
| Prnd | 2.280107919 | 5.12E-05 | up |
| Slc27a1 | 1.347165386 | 1.94E-11 | up |
| Vnn3 | 1.450661409 | 1.80E-20 | up |
| Nlrc4 | 1.485426827 | 1.41E-45 | up |
| Colgalt2 | 1.033947332 | 4.13E-06 | up |
| Mgat4a | 10.34429591 | 4.14E-04 | up |
| Chd5 | 1.070389328 | 1.80E-04 | up |
| Rbbp8nl | 1.959358016 | 4.83E-08 | up |
| Pla2g7 | 2.044394119 | 2.14E-04 | up |
| Trpa1 | 15.94763694 | 1.83E-191 | up |
| Slco1a6 | 3.678071905 | 1.20E-13 | up |
| Stau2 | 1.146609835 | 1.20E-17 | up |
| Snai3 | 1.35614381 | 8.52E-05 | up |
| Tor3a | 1.046542586 | 4.36E-06 | up |
| Lmcd1 | 1.698173111 | 4.62E-28 | up |
| Fgd3 | 1.231830906 | 1.54E-47 | up |
| Idi1 | 1.954631572 | 0 | up |
| Galm | 2.263034406 | 1.14E-18 | up |
| A530016L24Rik | 1.0489096 | 2.35E-08 | up |
| Olfml2b | 2.38466385 | 5.87E-07 | up |
| Ankrd44 | 2.050626073 | 5.70E-04 | up |
| Ksr2 | 11.03617361 | 2.08E-06 | up |
| Slc41a2 | 5.169925001 | 9.99E-19 | up |
| Tmem102 | 1.058893689 | 4.94E-04 | up |
| S100a7a | 1.366782331 | 5.71E-07 | up |
| B3glct | 1.235628248 | 1.08E-08 | up |
| Aim2 | 2.638981039 | 5.7485409596775E-311 | up |
| Kng2 | 5.74723393 | 2.51E-101 | up |
| Tnip3 | 1.030373649 | 9.56E-05 | up |
| Plcxd2 | 1.388083538 | 1.31E-88 | up |
| Gm13212 | 2.415608073 | 2.93E-05 | up |
| Zfp985 | 5.008736934 | 4.97E-70 | up |
| Ociad2 | 1.821029859 | 8.73E-06 | up |
| E2f6 | 15.15560884 | 3.26E-110 | up |
| Mfap5 | 10.34429591 | 4.14E-04 | up |
| Fbxo17 | 1.100136671 | 2.85E-16 | up |
| Slc7a8 | 3.662965013 | 2.59E-08 | up |
| Rab11fip5 | 1.854149134 | 2.51E-05 | up |
| Stbd1 | 3.058893689 | 2.62E-09 | up |
| Ifi27 | 1.91753784 | 8.08E-25 | up |
| Dlg3 | 6.807354922 | 8.48E-32 | up |
| Rcan2 | 4.754887502 | 4.97E-07 | up |
| Ctnnal1 | 1.403722186 | 4.21E-09 | up |
| Hpgds | 4.103691303 | 1.19E-200 | up |
| Arhgap40 | 2.459431619 | 9.15E-07 | up |
| Gm13043 | 3.751446181 | 6.31E-06 | up |
| Calcrl | 1.793549123 | 1.35E-05 | up |
| Gramd2 | 1.914270126 | 1.20E-10 | up |
| Gm5977 | 1.643432843 | 4.42E-05 | up |
| Grasp | 1.510961919 | 5.86E-05 | up |
| Cpb2 | 2.497499659 | 3.91E-14 | up |
| Ctsf | 2.222392421 | 3.63E-04 | up |
| Fzd2 | 1.217591435 | 4.21E-06 | up |
| Bcam | 1.298341275 | 4.10E-06 | up |
| Smpdl3a | 5.285402219 | 2.06E-10 | up |
| Jph1 | 1.230297619 | 2.32E-07 | up |
| Cysltr1 | 1.678071905 | 2.20E-22 | up |
| Fetub | 2.906890596 | 1.29E-05 | up |
| Ms4a4b | 2.184424571 | 1.03E-06 | up |
| Gm6763 | 10.666224 | 5.78E-05 | up |
| Bco1 | 3.169925001 | 1.05E-08 | up |
| Aqp9 | 4.754887502 | 4.97E-07 | up |
| Smoc1 | 3.906890596 | 8.67E-04 | up |
| Moap1 | 1.052096998 | 4.95E-04 | up |
| Ptges | 1.862496476 | 1.13E-04 | up |
| Gpr85 | 1.648288436 | 3.36E-14 | up |
| Lpin3 | 2.297680549 | 3.22E-08 | up |
| Ifi30 | 1.100194288 | 6.32E-15 | up |
| Fbxo36 | 1.392317423 | 3.08E-06 | up |
| Msmo1 | 1.613108806 | 0 | up |
| Aig1 | 1.950683747 | 1.55E-21 | up |
| Gm7694 | 1.402964667 | 1.41E-08 | up |
| Zscan4f | 1.286638295 | 7.28E-07 | up |
| 1700049E17Rik1 | 3.103279943 | 4.47E-11 | up |
| 9930111J21Rik1 | 10.32080055 | 7.94E-04 | up |
| Zfp600 | 1.274277236 | 1.75E-06 | up |
| Gm8909 | 1.448186828 | 3.29E-12 | up |
| 9030624G23Rik | 2.653114482 | 1.66E-04 | up |
| Eif4a3l1 | 2.334854269 | 1.35E-04 | up |
| Gm9040 | 11.33371443 | 1.42E-07 | up |
| Gm9045 | 10.72621816 | 5.78E-05 | up |
| Trim35 | 1.001522633 | 1.06E-74 | up |
| Nkain1 | 1.268105395 | 5.34E-20 | up |
| 2610301B20Rik | 2.038135129 | 2.50E-13 | up |
| Aldh3b1 | 1.468279521 | 1.77E-26 | up |
| Rgs10 | 3.906890596 | 8.68E-04 | up |
| Plpp3 | 1.371968777 | 4.96E-23 | up |
| Gnpda2 | 1.17948383 | 3.98E-14 | up |
| Gsto2 | 2.029747343 | 4.29E-06 | up |
| Micu2 | 1.155030786 | 1.37E-26 | up |
| Angel1 | 1.514573173 | 9.69E-04 | up |
| Atp2c2 | 3.906890596 | 8.67E-04 | up |
| Tmem121 | 5.523561956 | 2.03E-12 | up |
| Esam | 3.247927513 | 3.95E-04 | up |
| Gpx8 | 10.45121111 | 2.15E-04 | up |
| Clybl | 1.523561956 | 6.93E-06 | up |
| Fam83g | 5.266786541 | 3.64E-20 | up |
| Gm10499 | 1.375855153 | 1.81E-32 | up |
| Smyd3 | 1.079727192 | 4.97E-13 | up |
| Rsc1a1 | 13.85564717 | 2.13E-44 | up |
| 2210016F16Rik | 4.217230716 | 1.50E-21 | up |
| Iyd | 2.736965594 | 8.48E-04 | up |
| Megf10 | 2.339850003 | 3.18E-11 | up |
| Ddo | 2.415037499 | 4.91E-05 | up |
| 3830403N18Rik | 6.894817763 | 7.02E-34 | up |
| Eva1c | 2.678071905 | 1.55E-05 | up |
| Otud1 | 1.088809267 | 8.93E-07 | up |
| Fmnl2 | 1.527108115 | 5.42E-46 | up |
| Snx20 | 2.321928095 | 1.22E-11 | up |
| Zcchc24 | 1.584962501 | 1.51E-04 | up |
| Gcnt3 | 1.572787787 | 4.64E-28 | up |
| Psca | 3.064130337 | 1.30E-16 | up |
| Sting1 | 1.35614381 | 8.52E-05 | up |
| Veph1 | 1.788667509 | 5.75E-11 | up |
| Tm7sf2 | 1.250961574 | 6.78E-14 | up |
| Vgll3 | 1.268995964 | 1.81E-44 | up |
| Irak3 | 1.526545814 | 9.95E-10 | up |
| Krt80 | 1.622315915 | 1.26E-76 | up |
| Cyp2s1 | 1.062509201 | 5.76E-25 | up |
| Stra6l | 10.34429591 | 4.15E-04 | up |
| Exoc3l4 | 1.310787537 | 2.01E-08 | up |
| Stx11 | 1.47883415 | 4.90E-10 | up |
| Dynap | 2.839842775 | 1.81E-80 | up |
| Rilpl1 | 1.126719989 | 4.43E-05 | up |
| Tmem14a | 1.081529885 | 7.80E-05 | up |
| Faxc | 13.5980525 | 3.74E-37 | up |
| Abca6 | 1.432959407 | 2.88E-06 | up |
| 0610040J01Rik | 1.538976295 | 3.11E-19 | up |
| Cep112 | 2.722466024 | 9.05E-06 | up |
| Prss23 | 1.242360838 | 4.21E-06 | up |
| Msrb2 | 2.185866545 | 1.33E-11 | up |
| Rflnb | 1.680721484 | 5.29E-10 | up |
| Bcas1 | 2.857980995 | 2.25E-05 | up |
| Shisa4 | 1.40053793 | 1.08E-13 | up |
| Nuak1 | 1.034215715 | 2.73E-11 | up |
| Nav2 | 1.095208023 | 2.86E-27 | up |
| Aacs | 1.378345232 | 1.32E-111 | up |
| Mettl27 | 1.140481224 | 1.85E-05 | up |
| Gpr146 | 1.38466385 | 7.74E-04 | up |
| Tfcp2l1 | 1.388152836 | 1.81E-70 | up |
| Klb | 2.289506617 | 2.70E-06 | up |
| Akap12 | 1.064555535 | 3.17E-27 | up |
| Pcdhga3 | 10.906139 | 7.91E-06 | up |
| Pcdhga5 | 1.056480642 | 1.80E-04 | up |
| Pcdhga9 | 11.94763694 | 1.08E-11 | up |
| Igsf9 | 2.129283017 | 8.37E-05 | up |
| Pcdhb16 | 1.2410081 | 4.58E-05 | up |
| Nlrp4f | 4.544320516 | 4.48E-17 | up |
| Atp1a2 | 3.209453366 | 2.41E-07 | up |
| Rdh10 | 1.983143326 | 0 | up |
| Gm13306 | -5.190212016 | 2.24E-05 | down |
| Mroh2a | -2.188902676 | 8.92E-10 | down |
| Gm10488 | -10.34429591 | 5.42E-04 | down |
| Gm3776 | -1.748408526 | 2.01E-54 | down |
| Gm10639 | -2.024935194 | 3.73E-07 | down |
| Nhsl2 | -1.365191256 | 9.79E-19 | down |
| Gm14308 | -1.504934986 | 3.09E-12 | down |
| Gm17783 | -10.8917837 | 1.17E-05 | down |
| Zfp967 | -11.58965115 | 9.03E-09 | down |
| Smpdl3b | -1.275634443 | 6.59E-34 | down |
| Ccdc149 | -13.24376903 | 3.83E-28 | down |
| Spon2 | -1.144873629 | 1.38E-07 | down |
| Ugt2b34 | -1.654963642 | 6.64E-216 | down |
| Gm21293 | -12.40434329 | 1.14E-15 | down |
| Gm21451 | -1.155174994 | 7.50E-06 | down |
| Wtip | -13.15165083 | 2.10E-26 | down |
| Gldc | -1.632268215 | 5.83E-139 | down |
| Rcor2 | -1.068306013 | 4.31E-14 | down |
| Arrdc3 | -1.186576242 | 1.17E-42 | down |
| Gm40035 | -3.479667558 | 4.51E-11 | down |
| Gm40364 | -11.97978243 | 1.25E-11 | down |
| Gm40369 | -3.899921041 | 5.36E-39 | down |
| Slc17a3 | -3.827819025 | 6.27E-15 | down |
| Cth | -1.156429159 | 2.78E-18 | down |
| Ltbp4 | -5.581953751 | 2.28E-123 | down |
| Prkab2 | -1.208108195 | 2.86E-10 | down |
| Gm45988 | -12.2366122 | 6.13E-14 | down |
| Prr5 | -12.64385619 | 1.46E-18 | down |
| Abca4 | -2.252387162 | 3.42E-10 | down |
| Adam15 | -1.168719239 | 7.92E-41 | down |
| Adamts1 | -1.444784843 | 2.35E-28 | down |
| LOC115486519 | -1.959358016 | 3.48E-04 | down |
| Adra1a | -10.64385619 | 8.09E-05 | down |
| Aldh1a1 | -1.919657892 | 0 | down |
| Aqp1 | -3.566346823 | 5.02E-30 | down |
| LOC118567439 | -4.063325935 | 3.76E-45 | down |
| LOC118567843 | -1.283700255 | 7.19E-04 | down |
| LOC118568050 | -1.301012086 | 2.65E-11 | down |
| LOC118568339 | -1.960242291 | 9.07E-04 | down |
| Atp7b | -1.203391459 | 1.62E-58 | down |
| Cbs | -1.480805269 | 4.60E-25 | down |
| Cd1d1 | -1.069041644 | 5.97E-05 | down |
| Cideb | -2.245132963 | 7.06E-25 | down |
| Ackr3 | -1.011404763 | 2.78E-05 | down |
| Col18a1 | -1.430061106 | 0 | down |
| Col6a1 | -3.841302254 | 2.79E-09 | down |
| Cpeb1 | -12.34429591 | 4.34E-15 | down |
| Cys1 | -1.249978253 | 3.33E-05 | down |
| Crp | -2 | 9.32E-13 | down |
| Dlg4 | -1.700439718 | 4.12E-10 | down |
| Ebf1 | -3.093976148 | 3.24E-19 | down |
| Ecm1 | -1.088227183 | 3.69E-10 | down |
| Efnb1 | -1.055495113 | 8.54E-09 | down |
| Emp2 | -5.196397213 | 1.55E-27 | down |
| Rem2 | -1.807354922 | 1.91E-04 | down |
| Fcgrt | -1.707819249 | 9.97E-05 | down |
| Fjx1 | -1.167530486 | 3.35E-14 | down |
| Gjb1 | -1.94096453 | 6.37E-40 | down |
| Gsta1 | -2.173940724 | 1.59E-153 | down |
| Gsta2 | -1.478848762 | 4.68E-05 | down |
| Gstm1 | -1.486463867 | 2.06E-55 | down |
| Gstm2 | -1.523561956 | 1.06E-11 | down |
| Gstm4 | -1.911463325 | 3.35E-08 | down |
| Gstm5 | -1.390459477 | 1.59E-06 | down |
| Hap1 | -3.10433666 | 7.32E-08 | down |
| Hey1 | -1.777607579 | 7.20E-05 | down |
| Hipk2 | -1.638083805 | 2.92E-102 | down |
| Hs3st1 | -2.153983458 | 1.61E-45 | down |
| Hspg2 | -1.042187071 | 5.79E-52 | down |
| Il12rb2 | -1.024919545 | 1.37E-05 | down |
| Il6ra | -1.273018494 | 3.91E-17 | down |
| Kif12 | -1.352516415 | 7.15E-09 | down |
| Lgals4 | -1.317615102 | 7.63E-10 | down |
| Lhx1 | -2.432959407 | 3.36E-04 | down |
| Lmo4 | -1.045692091 | 1.11E-31 | down |
| Kirrel | -1.026855492 | 5.10E-71 | down |
| Kcnh7 | -1.204726239 | 3.72E-14 | down |
| Acot4 | -13.98370619 | 3.70E-47 | down |
| Bmf | -1.025237291 | 5.05E-07 | down |
| Mycn | -2.550197083 | 2.83E-06 | down |
| Notch2 | -1.131911676 | 1.15E-127 | down |
| Npc1 | -3.202267274 | 9.00E-238 | down |
| Nrp1 | -1.091268058 | 1.45E-21 | down |
| Pde7a | -1.009554307 | 6.26E-22 | down |
| Pla2g4a | -2.145540843 | 4.44E-88 | down |
| Prl2c2 | -1.425893865 | 0 | down |
| Ash1l | -1.046924981 | 1.10E-113 | down |
| Ptgfrn | -1.326002799 | 2.07E-170 | down |
| Rbp1 | -1.380821784 | 4.31E-08 | down |
| S100a4 | -1.544005826 | 3.30E-27 | down |
| Cx3cl1 | -1.770518154 | 1.76E-15 | down |
| Slc2a2 | -1.2410081 | 4.94E-11 | down |
| Slc3a1 | -2.043216176 | 9.26E-06 | down |
| Snap25 | -2 | 1.45E-07 | down |
| Sod3 | -2.906890596 | 2.22E-05 | down |
| Sox13 | -1 | 3.76E-10 | down |
| Rbms3 | -1.166014787 | 4.70E-04 | down |
| Ggt7 | -2.584962501 | 5.00E-04 | down |
| Sprr1a | -1.097173503 | 3.78E-99 | down |
| Sprr2h | -2.863498 | 6.54E-21 | down |
| Gm4767 | -1.34019101 | 1.89E-04 | down |
| Kbtbd7 | -3.365810156 | 3.28E-17 | down |
| Spsb4 | -3.930737338 | 3.78E-13 | down |
| Arhgap29 | -1.841302254 | 1.26E-04 | down |
| Slc43a2 | -1.094563002 | 4.81E-14 | down |
| Psd4 | -1.683366205 | 3.10E-22 | down |
| Miga1 | -6.475733431 | 3.44E-24 | down |
| Aldh1l2 | -1.201633861 | 1.81E-10 | down |
| Agap2 | -2.638600464 | 4.89E-20 | down |
| Tead2 | -1.379152964 | 5.79E-27 | down |
| Spns2 | -2.289172002 | 1.47E-115 | down |
| Prr15l | -1.614709844 | 1.98E-04 | down |
| Engase | -1.250543462 | 2.87E-04 | down |
| Mlh3 | -1.150491627 | 3.24E-19 | down |
| Lysmd1 | -1 | 1.76E-04 | down |
| Ugt2b5 | -2.934112064 | 1.28E-17 | down |
| Dpysl3 | -1.686382819 | 5.70E-31 | down |
| Vldlr | -1.286738666 | 6.72E-22 | down |
| Zfp61 | -2.362570079 | 3.41E-05 | down |
| Ikzf2 | -2.874469118 | 4.25E-04 | down |
| Gca | -1.203634363 | 2.37E-41 | down |
| 4932438A13Rik | -1.113836237 | 3.92E-55 | down |
| Fcrl1 | -1.253118937 | 1.69E-09 | down |
| Smg5 | -1.015283345 | 1.09E-131 | down |
| Amigo1 | -1.160464672 | 3.46E-04 | down |
| Clcc1 | -1.061983131 | 3.29E-29 | down |
| Acnat1 | -1.315418067 | 1.31E-30 | down |
| Aldob | -2.159545856 | 1.99E-31 | down |
| 5730409E04Rik | -13.16741815 | 1.08E-26 | down |
| Ugt2b36 | -2.35453276 | 8.12E-23 | down |
| Synm | -1.14974712 | 6.35E-04 | down |
| P2ry6 | -1.914270126 | 2.15E-05 | down |
| Tmem184c | -1 | 5.40E-29 | down |
| C2cd4c | -1.216317907 | 2.47E-08 | down |
| Rtn4rl1 | -2.380423744 | 1.32E-19 | down |
| Gpc6 | -1.197939378 | 1.87E-04 | down |
| Tenm4 | -2.182765347 | 1.51E-134 | down |
| Rasgrp3 | -1.047068254 | 7.81E-16 | down |
| Slc16a12 | -1.679690789 | 9.32E-43 | down |
| Srp54a | -1.149652606 | 1.88E-04 | down |
| Tlr2 | -1.56828376 | 6.90E-07 | down |
| Zfp385b | -4.169925001 | 2.00E-04 | down |
| Bbs12 | -1.247092862 | 1.65E-11 | down |
| Ppm1l | -1.164341428 | 2.06E-49 | down |
| Ugt2b35 | -3.308752706 | 1.29E-39 | down |
| Hdx | -4.807354922 | 4.59E-07 | down |
| Tox | -1.149612546 | 2.43E-15 | down |
| Cadm4 | -1.434937057 | 2.49E-05 | down |
| Bahcc1 | -1.191986764 | 1.51E-58 | down |
| Trim36 | -1.754887502 | 2.69E-05 | down |
| Ndrg2 | -1.498681159 | 4.60E-34 | down |
| H2bc21 | -1.932885804 | 7.48E-29 | down |
| Slc17a4 | -1.365649472 | 1.17E-04 | down |
| Rnf152 | -1.440572591 | 2.28E-04 | down |
| Fry | -2 | 1.45E-07 | down |
| Itpkb | -6.727920455 | 4.61E-29 | down |
| Zscan30 | -1.195186051 | 6.41E-05 | down |
| Acot11 | -3.528378972 | 1.68E-07 | down |
| Arhgap32 | -3.523561956 | 5.97E-05 | down |
| Egfl7 | -1.669851398 | 3.13E-06 | down |
| Col27a1 | -1.182789219 | 3.16E-48 | down |
| Raet1e | -1.691647266 | 4.14E-16 | down |
| Vgf | -1.992305608 | 2.29E-19 | down |
| Zcwpw1 | -3.192894777 | 6.61E-38 | down |
| Ugt1a7c | -1.637240721 | 2.25E-211 | down |
| Akr1c19 | -1.539616924 | 2.61E-48 | down |
| Maml3 | -1.404390255 | 1.45E-11 | down |
| H1f2 | -1.619314006 | 1.03E-19 | down |
| Bbs1 | -2.378511623 | 5.46E-04 | down |
| Ncald | -1.813988014 | 2.64E-10 | down |
| Zfp973 | -11.33147682 | 2.38E-07 | down |
| Car5b | -1.740316804 | 2.36E-36 | down |
| Ptbp2 | -1.083081741 | 6.07E-17 | down |
| Tspan5 | -1.222392421 | 1.44E-14 | down |
| Pkp3 | -1.348400306 | 3.15E-16 | down |
| Slc2a5 | -1.456064768 | 2.49E-14 | down |
| Raet1d | -1.510788137 | 1.94E-14 | down |
| Crtap | -14.43827206 | 8.79E-65 | down |
| Psrc1 | -1.607158247 | 2.09E-10 | down |
| Ttyh1 | -1.387023123 | 8.52E-07 | down |
| Fmnl1 | -1.470846408 | 1.64E-37 | down |
| Rangrf | -2.146355308 | 2.25E-14 | down |
| Chst11 | -11.39874369 | 6.43E-08 | down |
| Pcbp3 | -1.691877705 | 1.94E-07 | down |
| Ugt1a8 | -10.72877085 | 8.09E-05 | down |
| Trim71 | -4.807354922 | 4.59E-07 | down |
| Sv2a | -4.938599455 | 1.59E-22 | down |
| Gprc5b | -1.584962501 | 1.69E-10 | down |
| Camk2n1 | -1.313157885 | 1.88E-18 | down |
| Rnf113a2 | -2 | 9.08E-23 | down |
| Prxl2b | -10.55074679 | 1.53E-04 | down |
| Cul7 | -1.142444265 | 7.36E-11 | down |
| H60b | -1.350098768 | 5.57E-11 | down |
| Dnajb4 | -1.154415628 | 4.93E-24 | down |
| ccdc198 | -2.95419631 | 1.29E-05 | down |
| Ift80 | -1.014587536 | 2.77E-18 | down |
| Smim6 | -1.415037499 | 1.08E-04 | down |
| Flnc | -1.720349781 | 0 | down |
| Dtwd2 | -1.174497731 | 2.18E-04 | down |
| Dipk2a | -1.011333091 | 3.79E-23 | down |
| Scnm1 | -1.247927513 | 2.35E-08 | down |
| Nt5dc2 | -1.043604769 | 7.24E-22 | down |
| Zfp619 | -1.201633861 | 4.68E-04 | down |
| Prxl2a | -1.810137363 | 4.12E-46 | down |
| Dnajb14 | -1.014746585 | 1.48E-21 | down |
| Ggt6 | -1.026472211 | 8.72E-05 | down |
| Zfp251 | -1.179323699 | 8.67E-08 | down |
| Syce2 | -1.872244453 | 1.65E-20 | down |
| Bdh1 | -1 | 1.67E-05 | down |
| Mfsd8 | -1.349334252 | 2.98E-11 | down |
| Zc2hc1c | -2.273018494 | 3.57E-04 | down |
| Ces2g | -1.699979161 | 2.24E-63 | down |
| Atxn7l2 | -1.473931188 | 7.42E-09 | down |
| Skida1 | -1.847996907 | 5.06E-04 | down |
| Pear1 | -1.922981978 | 2.25E-192 | down |
| Camkmt | -1.035046947 | 2.20E-05 | down |
| Fam151b | -1.509013647 | 9.45E-06 | down |
| Slc25a27 | -1.124328135 | 2.17E-05 | down |
| Phf19 | -12.64385619 | 1.46E-18 | down |
| Paqr5 | -1.890375509 | 7.56E-15 | down |
| Slc6a19 | -2.410933101 | 1.40E-15 | down |
| 4930432K21Rik | -1.862496476 | 4.92E-08 | down |
| Mageh1 | -13.2137118 | 1.46E-27 | down |
| Nudt16 | -10.55074679 | 1.53E-04 | down |
| Sesn3 | -1.018262206 | 4.58E-12 | down |
| Them4 | -1.357552005 | 4.50E-17 | down |
| Tcam1 | -1.27897595 | 3.37E-10 | down |
| Gon4l | -1.018118371 | 5.84E-99 | down |
| Atp6v0e2 | -1.577545029 | 1.22E-14 | down |
| Lrmda | -10.81378119 | 2.24E-05 | down |
| Tmem181a | -1.595740275 | 1.46E-23 | down |
| Tmem200a | -4.087462841 | 9.19E-08 | down |
| Heg1 | -1.099535674 | 7.25E-04 | down |
| Ulbp1 | -2.340946949 | 1.55E-132 | down |
| Zfp449 | -10.96578428 | 6.16E-06 | down |
| Filip1l | -1.196969824 | 3.95E-25 | down |
| Ttc30a1 | -1.599243529 | 1.05E-15 | down |
| Trim2 | -1.473200525 | 3.14E-43 | down |
| Castor2 | -1.303392143 | 5.50E-07 | down |
| Gpr63 | -10.8917837 | 1.17E-05 | down |
| Tnxb | -1.547487795 | 1.10E-45 | down |
| Pcdhgc4 | -3.052708834 | 4.76E-13 | down |
| Pcdhga7 | -11.13570929 | 1.68E-06 | down |
| Pard6g | -10.55074679 | 1.53E-04 | down |
| Deptor | -1.828233652 | 3.76E-16 | down |
| Tlcd4 | -1.448460501 | 1.89E-25 | down |
| Tiparp | -1.096302138 | 9.16E-24 | down |

**Supplementary Table 2: Sequences of siRNAs**

| Genes | Species | Sequences (5′-3′) |
| --- | --- | --- |
| *siSYT7#1* | human | ACGAGACCTTCCTCTTTGA |
| *siSYT7#2* | human | GTGAGAAGAAGGCTATCAA |
| *siSYT7#3* | human | GAGTCCTTCGCCTTCGATA |
| *siVAPB#1* | human | CCAACAGCGGAATCATCGA |
| *siVAPB#2* | human | ACAGCACCACGTAGGTACT |
| *siVAPB#3* | human | CCGATGTTGTCACCACCAA |
| *siSTARD3#1* | human | GCAAGACGTTTATCCTGAA |
| *siSTARD3#2* | human | CGGCCACCATGTTTGAATT |
| *siSTARD3#3* | human | TCTGGATCATCGAACTGAA |
| *siOSBP#1* | human | GCACCATGCTGAGTCCAAA |
| *siOSBP#2* | human | GGAGAATACTGGGAGTGTA |
| *siOSBP#3* | human | GAACCAGAATACCATACAA |
| *siLDLR#1* | human | GGACAGATATCATCAACGA |
| *siLDLR#2* | human | GGCGTCTCTTCCTATGACA |
| *siLDLR#3* | human | GGGTCTTCCTTCTATGGAA |
| *siNPC1#1* | human | GCACCAGGTTCTTGACTTA |
| *siNPC1#2* | human | GCCATCATGTTTCTATATA |
| *siNPC1#3* | human | CCATGTTCCTTTCGGATAA |
| *siLIMA1#1* | human | GGTTAAGAGTGAGGTTCAA |
| *siLIMA1#2* | human | GGAGAATGAGAATCTTGTA |
| *siLIMA1#3* | human | GGTCGAGTTTTGTAGACAA |
| *siNPC2#1* | human | CTACCTGAATAAACTACCA |
| *siNPC2#2* | human | AGTGGCAACTTCAGGATGA |
| *siNPC2#3* | human | GCCCTATCCAAAAAGACAA |
| *siNPC1L1#1* | human | GTTGCACGTTGCAATGAGT |
| *siNPC1L1#2* | human | CTTTGAAGGTTCTATCAAA |
| *siNPC1L1#3* | human | CCATGGGCTTCTTCTCCTA |
| *siORP2#1* | human | GGACCGGCAAACCATTTAA |
| *siORP2#2* | human | GGAGAAACGTATGAATTAA |
| *siORP2#3* | human | AGAACGGAATTCAGAAACA |
| *siORP1L#1* | human | GCCGGATTCTGAAAGTGTA |
| *siORP1L#2* | human | CTGCCACGTTTGACGCTTA |
| *siORP1L#3* | human | AGATGACCTTGGATTTAGA |
| *siIDOL#1* | human | GAGACGCACGCATTCTACA |
| *siIDOL#2* | human | GCTCTACCGAGCGATAACA |
| *siIDOL#3* | human | GGCGACTGGGAATCATAGA |
| *siORP5#1* | human | GGAGAACGATGCATTCTCA |
| *siORP5#2* | human | GTCAGCTGTTCATTAACCA |
| *siORP5#3* | human | GGGACGTGTTTATCAAGGA |

**Supplementary Table 3:** **Primers used for gene amplification.**

| Genes | Species | Primer sequences (5′-3′) |
| --- | --- | --- |
| *ACTB* | human | F: CATGTACGTTGCTATCCAGGC |
|  |  | R: CTCCTTAATGTCACGCACGAT |
| *IFIT1* | human | F: AGAAGCAGGCAATCACAGAAAA |
|  |  | R: CTGAAACCGACCATAGTGGAAAT |
| *IFIT2* | human | F: GACACGGTTAAAGTGTGGAGG |
|  |  | R: TCCAGACGGTAGCTTGCTATT |
| *IFI44* | human | F: ATGGCAGTGACAACTCGTTTG |
|  |  | R: TCCTGGTAACTCTCTTCTGCATA |
| *NPC1* | human | F: ATGACCGCTCGCGGCC |
|  |  | R: CTAGAAATTTAGAAGCCGTTGCTCTGT |
| *SREBF2* | human | F: AACGGTCATTCACCCAGGTC |
|  |  | R: GGCTGAAGAATAGGAGTTGCC |
| *FASN* | human | F: AAGGACCTGTCTAGGTTTGATGC |
|  |  | R: TGGCTTCATAGGTGACTTCCA |
| *SQLE* | human | F: GGCATTGCCACTTTCACCTAT |
|  |  | R: GGCCTGAGAGAATATCCGAGAAG |
| *HMGCR* | human | F: TGATTGACCTTTCCAGAGCAAG |
|  |  | R: CTAAAATTGCCATTCCACGAGC |
| *ACACA* | human | F: ATGTCTGGCTTGCACCTAGTA |
|  |  | R: CCCCAAAGCGAGTAACAAATTCT |
| *DHCR7* | human | F: GCAGGGGTTGTGAACAAGTAT |
|  |  | R: GAGACGGCATAGCCAAGGAT |
| *IFNB1* | human | F: GCTTGGATTCCTACAAAGAAGCA |
|  |  | R: ATAGATGGTCAATGCGGCGTC |
| *SYT7* | human | F: TCATCACCGTCAGCCTTAGC |
|  |  | R: TCTTGTAGCGTTTGCCCAGTT |
| *VAPB* | human | F: AGATGGACTGCGGATGAGGAA |
|  |  | R: CAGTTGGGGCTAATGCTGAAA |
| *STARD3* | human | F: GGGCCATCTCTGATGTCCG |
|  |  | R: GCCTGTGTTGGTATTCAGTTCG |
| *OSBP* | human | F: CTACCAGCGGCGATGGTTC |
|  |  | R: ACCACGGCAGGTATGTCTCAT |
| *LDLR* | human | F: ACCAACGAATGCTTGGACAAC |
|  |  | R: ACAGGCACTCGTAGCCGAT |
| *LIMA1* | human | F: GACTCCCAGGTTAAGAGTGAGG |
|  |  | R: TTGCAGGTGCCTGAAACTTCT |
| *NPC2* | human | F: CAAAGGACAGTCTTACAGCGT |
|  |  | R: GGATAGGGCAGTTAATTCCACTC |
| *NPC1L1* | human | F: AGAGTGAGCCTTACACAACCA |
|  |  | R: GCAGGACACGTTGGAGAGT |
| *ORP2* | human | F: CAGAGGCAAATCAGAAAGTCACG |
|  |  | R: TCCCCAGTTTTCCCAATCCTA |
| *ORP1L* | human | F: CATACTTACCTCATCCACAAGGC |
|  |  | R: CAAGGTCATCTCGCACTAATTCA |
| *IDOL* | human | F: GCAGGCGACTGGGAATCATAG |
|  |  | R: CGGTTTCTCAGGTTTAGCCAT |
| *ORP5* | human | F: CTCCGAGTCAGATGGTCGC |
|  |  | R: TGCCCAGTCTCAGTAGGCT |
| *Actb* | mouse | F: GTGACGTTGACATCCGTAAAGA |
|  |  | R: GCCGGACTCATCGTACTCC |
| *Ifit1* | mouse | F: ATCGCGTAGACAAAGCTCTTC |
|  |  | R: GTTTCGGGATGTCCTCAGTTG |
| *Ifit2* | mouse | F: CTGGGGAAACTATGCTTGGGT |
|  |  | R: ACTCTCTCGTTTTGGTTCTTGG |
| *Ifi44* | mouse | F: ATGCTCCAACTGACTGCTCG |
|  |  | R: ACAGCAATGCCTCTTGTCTTT |
| *Sting* | mouse | F: TATACCTCAGTTGGATGTTTGGC |
|  |  | R: CTGGAGTCAAGCTCTGAAGGC |
| *Ifnb1* | mouse | F: AGCTCCAAGAAAGGACGAACA |
|  |  | R: GCCCTGTAGGTGAGGTTGAT |
| *Cxcl10* | mouse | F: GTGGCATTCAAGGAGTACCTC |
|  |  | R: TGATGGCCTTCGATTCTGGATT |
| H1N1-HA |  | F: CCCGGAAATAGCAGAAAGACCCAAAGTA |
|  |  | R: GCCGGACCCAAAGCCTCTACTCAGT |
| VSV-G |  | F: CAAGTCAAAATGCCCAAGAGTCACA |
|  |  | R: TTTCCTTGCATTGTTCTACAGATGG |
| EMCV-1C |  | F: CCGCGATGATGAAGGGCAAG |
|  |  | R: CGGGCATCCTGGTGGGTAAGT |
| HSV-1 ICP27 |  | F: TTTCTCCAGTGCTACCTGAAGG |
|  |  | R: TCAACTCGCAGACACGACTCG |
