# Supplementary figures and images for: Phytochemical alkaloids orchestrate immunometabolism against viral infections
Source: Natl Sci Rev. 2025 Jun 16;12(9):nwaf190. doi: 10.1093/nsr/nwaf190 (PMC12416277; doi:10.1093/nsr/nwaf190)

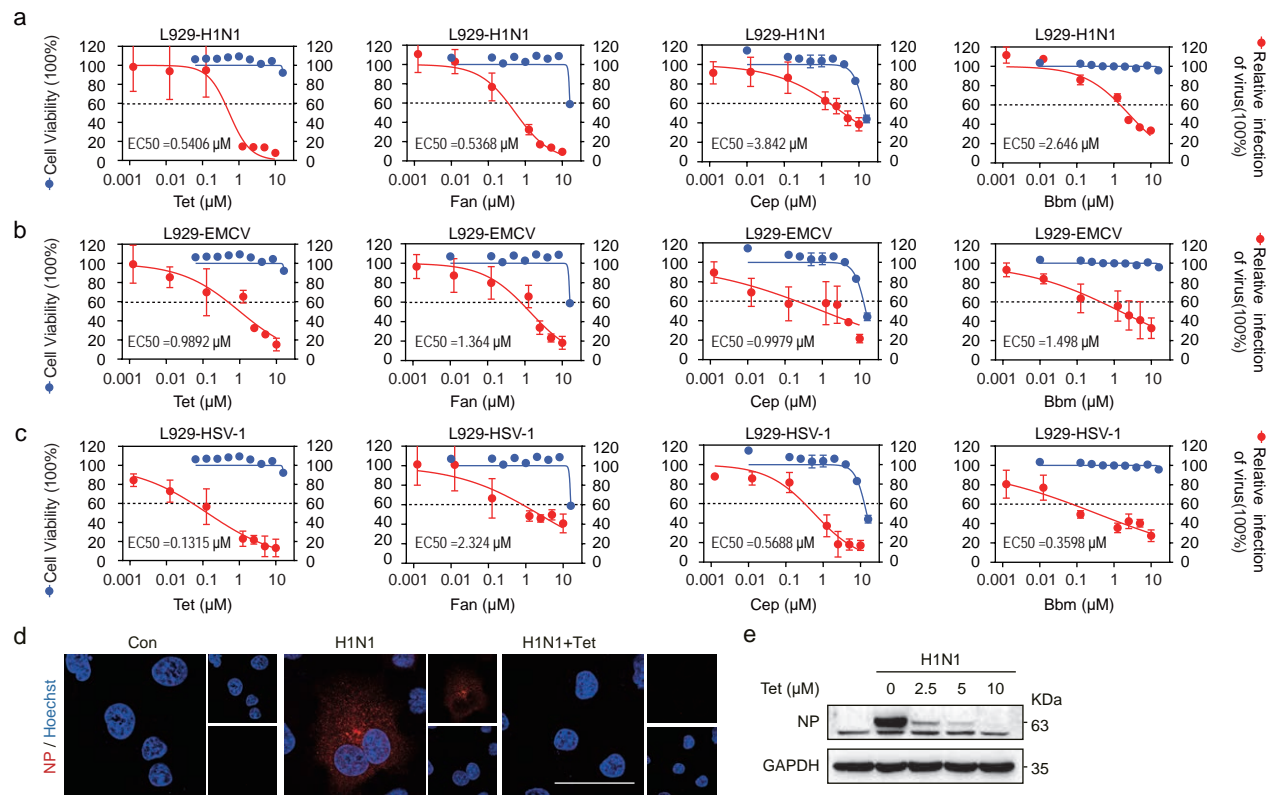

Supplement: nwaf190_Supplemental_Files [file nwaf190_supplemental_files.zip › [NSR_MS-2024-450.R2] Fig S2.20250424.pdf]

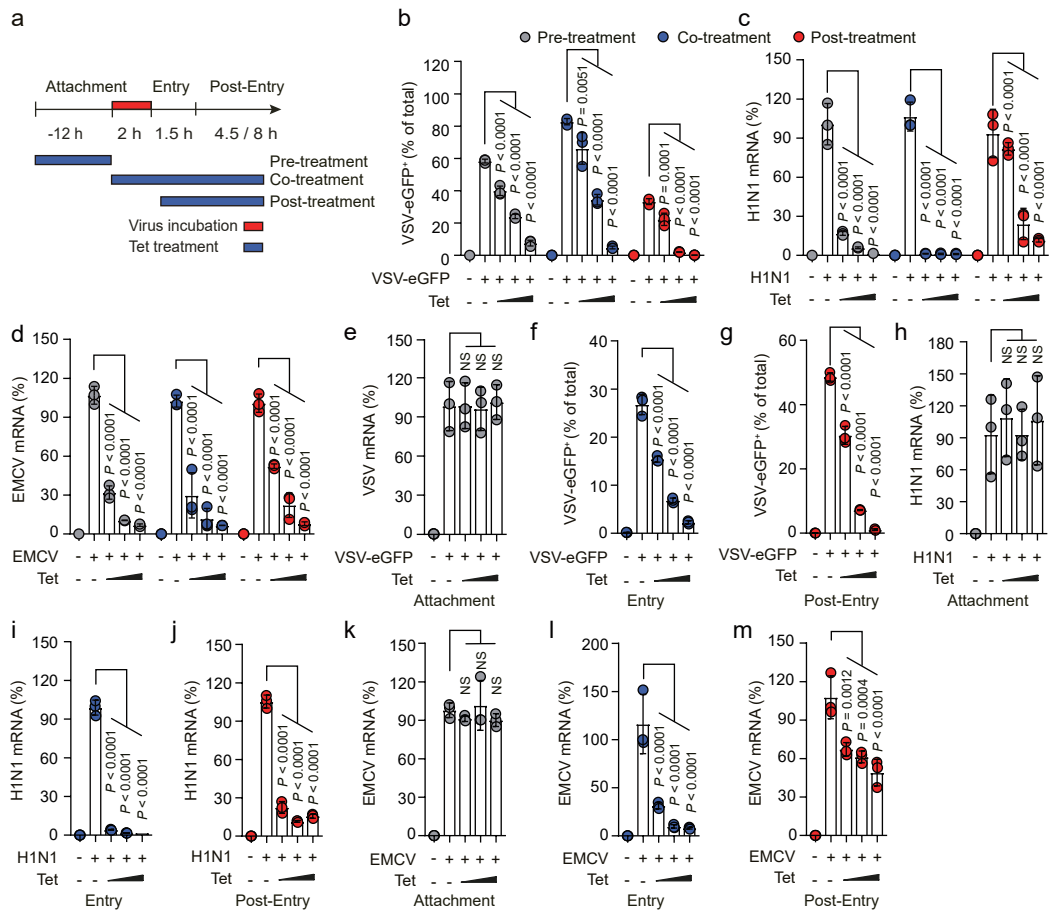

Supplement: nwaf190_Supplemental_Files [file nwaf190_supplemental_files.zip › [NSR_MS-2024-450.R2] Fig S3.20250424.pdf]

a

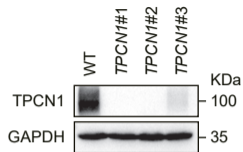

b

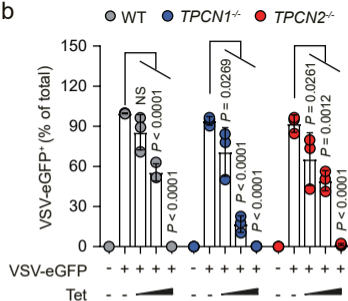

c

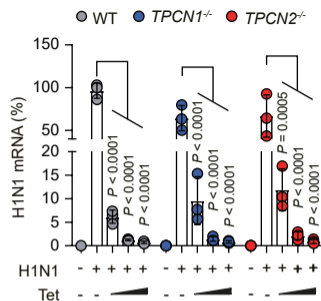

d

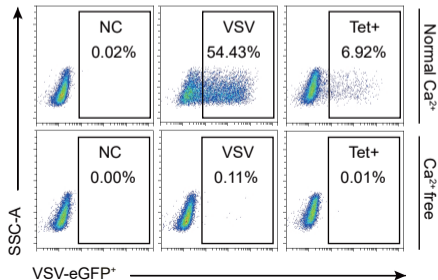

e

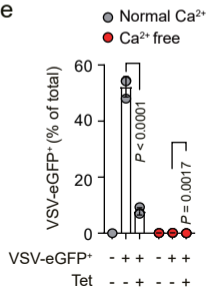

f

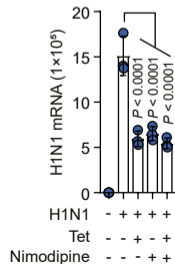

Supplement: nwaf190_Supplemental_Files [file nwaf190_supplemental_files.zip › [NSR_MS-2024-450.R2] Fig S4.20250424.pdf]

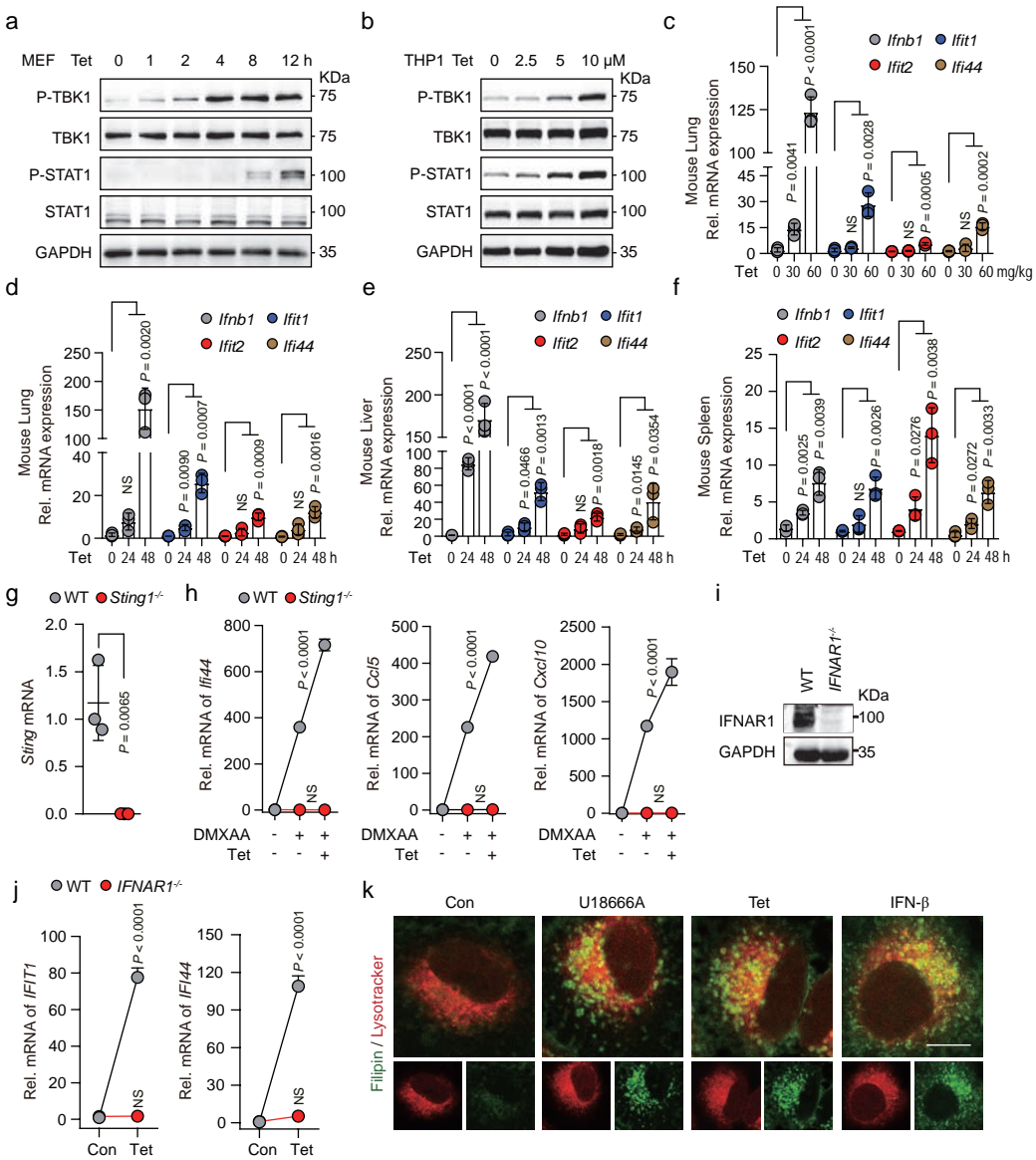

Supplement: nwaf190_Supplemental_Files [file nwaf190_supplemental_files.zip › [NSR_MS-2024-450.R2] Fig S5.20250424.pdf]

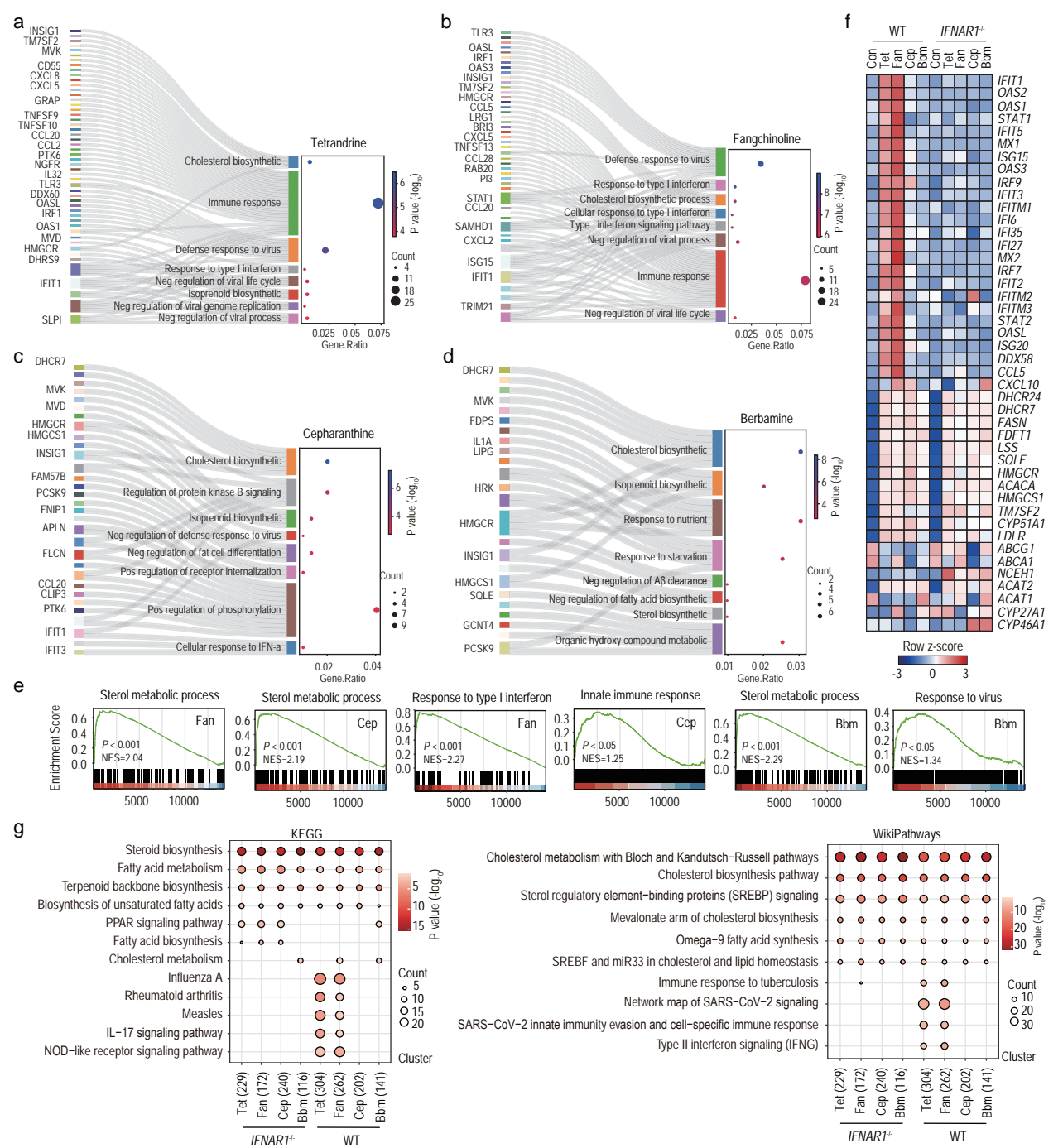

Supplement: nwaf190_Supplemental_Files [file nwaf190_supplemental_files.zip › [NSR_MS-2024-450.R2] Fig S6.20250424.pdf]

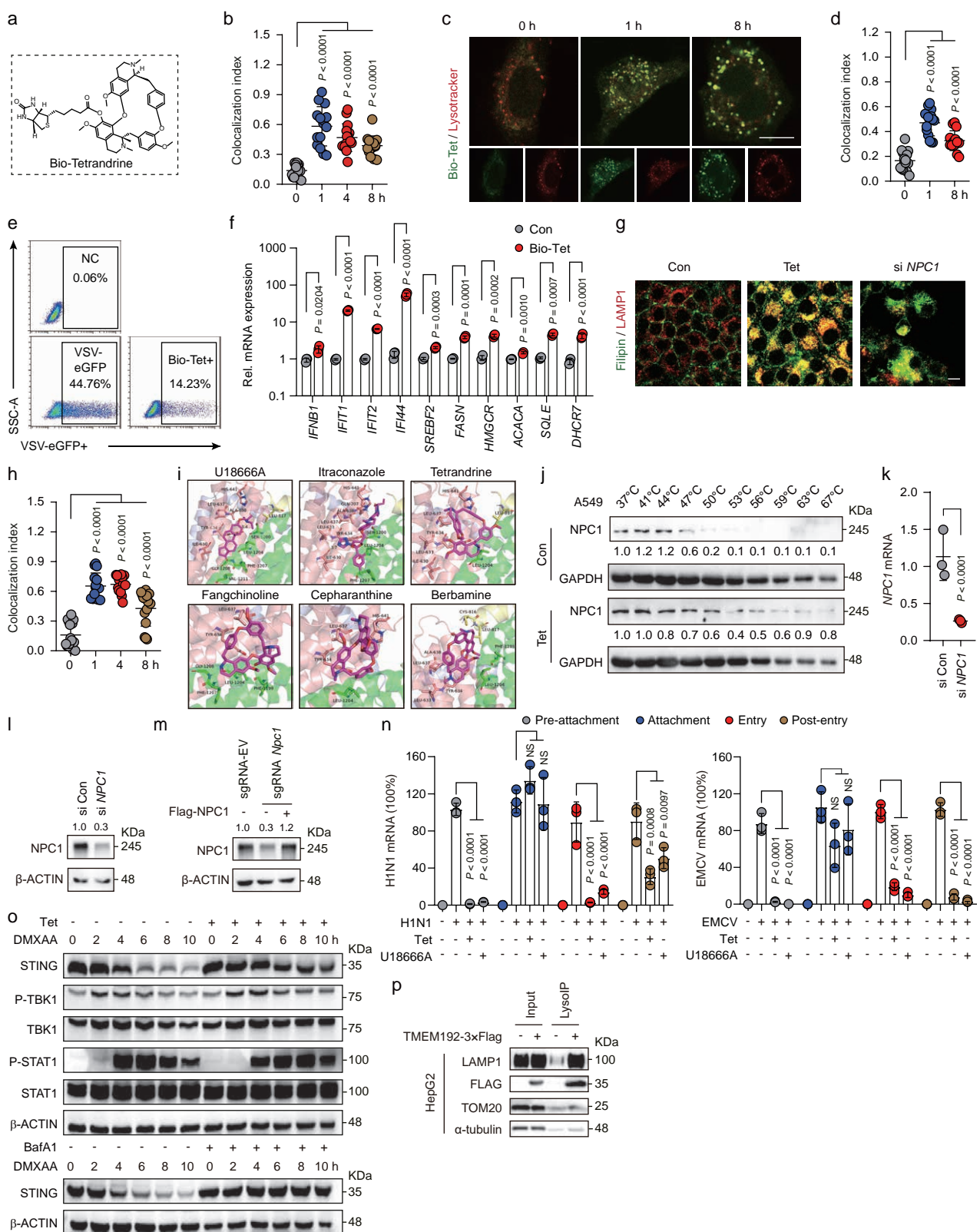

Supplement: nwaf190_Supplemental_Files [file nwaf190_supplemental_files.zip › [NSR_MS-2024-450.R2] Fig S7.20250424.pdf]
