# Supplementary material for: Phytochemical alkaloids orchestrate immunometabolism against viral infections
Source: Natl Sci Rev. 2025 Jun 16;12(9):nwaf190. doi: 10.1093/nsr/nwaf190 (PMC12416277; doi:10.1093/nsr/nwaf190)
Supplement: nwaf190_Supplemental_Files [file [NSR_MS-2024-450.R2] Fig S1.20250424.pdf › [NSR_MS-2024-450.R2] Fig S1.20250424.pdf]

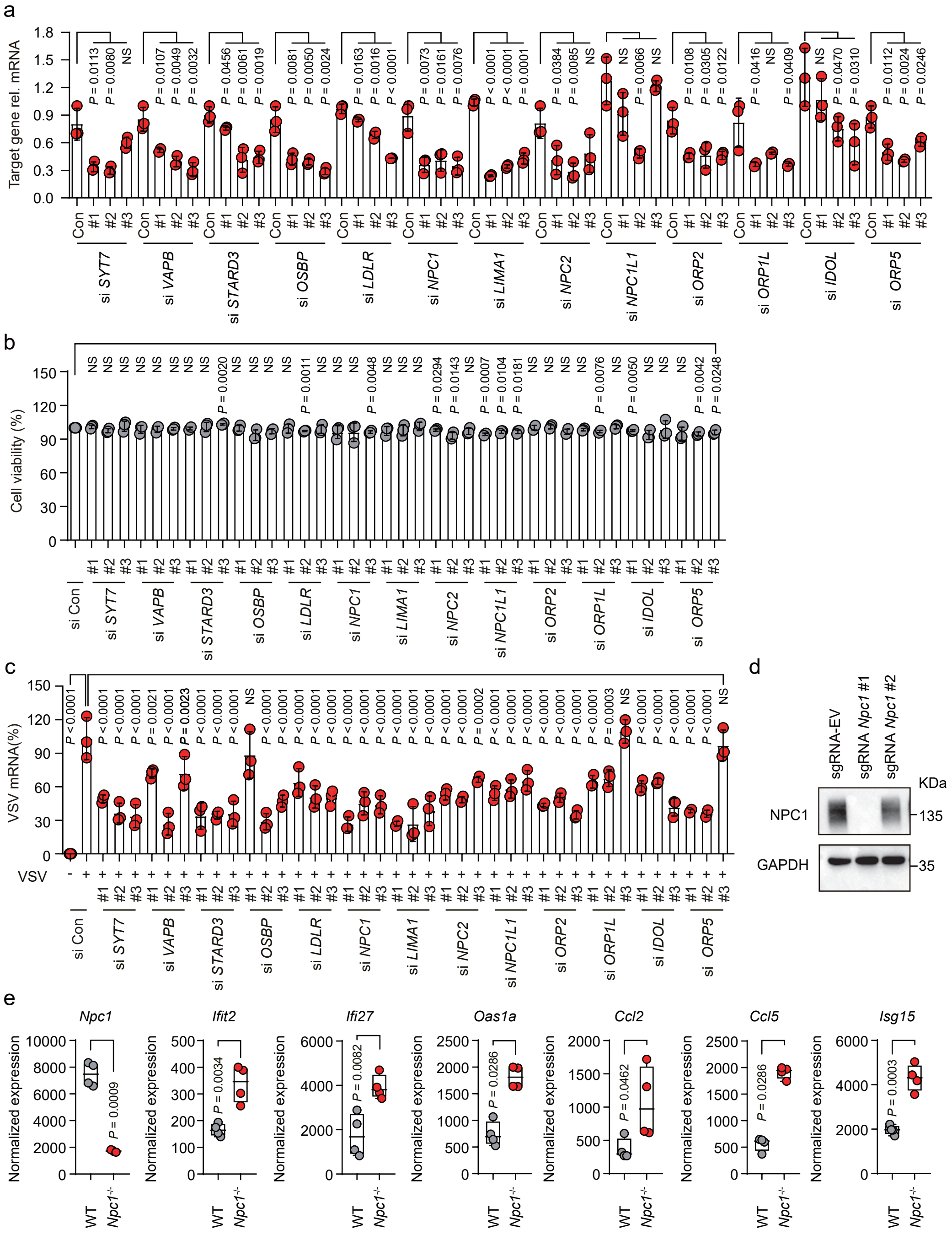
a
Target gene rel. mRNA
si SYT7
si VAPB
si STARD3
si OSBP
si LDLR
si NPC1
si LIMA1
si NPC2
si NPC1L1
si ORP2
si ORP1L
si IDOL
si ORP5
Con #1 #2 #3
b
Cell viability (%)
si Con
c
VSV mRNA(%)
VSV
d
sgRNA-EV
sgRNA Npc1 #1
sgRNA Npc1 #2
KDa
NPC1
GAPDH
135
35
e
Npc1
Ifit2
Ifi27
Oas1a
Ccl2
Ccl5
Isg15
Normalized expression
WT
Npc1-/-
P = 0.0009
P = 0.0034
P = 0.0082
P = 0.0286
P = 0.0462
P = 0.0286
P = 0.0003
